# Comparative Transcriptome Analysis Reveals Adaptive Evolution of *Notopterygium incisum* and *Notopterygium* *franchetii*, Two High-Alpine Herbal Species Endemic to China

**DOI:** 10.3390/molecules22071158

**Published:** 2017-07-11

**Authors:** Yun Jia, Mi-Li Liu, Ming Yue, Zhe Zhao, Gui-Fang Zhao, Zhong-Hu Li

**Affiliations:** Key Laboratory of Resource Biology and Biotechnology in Western China, Ministry of Education, College of Life Sciences, Northwest University, Xi’an 710069, China; jy878683@163.com (Y.J.); ml_i97@163.com (M.-L.L.); yueming@nwu.edu.cn (M.Y.); xiaoxiaohaizi1201@163.com (Z.Z.); gfzhao@nwu.edu.cn (G.-F.Z)

**Keywords:** adaptive evolution, microsatellite marker, *Notopterygium*, transcriptome, positive selection

## Abstract

The extreme conditions (e.g., cold, low oxygen, and strong ultraviolet radiation) of the high mountains provide an ideal natural laboratory for studies on speciation and the adaptive evolution of organisms. Up to now, few genome/transcriptome-based studies have been carried out on how plants adapt to conditions at extremely high altitudes. *Notopterygium incisum* and *Notopterygium* *franchetii* (*Notopterygium*, Apiaceae) are two endangered high-alpine herbal plants endemic to China. To explore the molecular genetic mechanisms of adaptation to high altitudes, we performed high-throughput RNA sequencing (RNA-seq) to characterize the transcriptomes of the two species. In total, more than 130 million sequence reads, 81,446 and 63,153 unigenes with total lengths of 86,924,837 and 62,615,693 bp, were generated for the two herbal species, respectively. OrthoMCL analysis identified 6375 single-copy orthologous genes between *N. incisum* and *N. franchetii*. In total, 381 positively-selected candidate genes were identified for both plants by using estimations of the non-synonymous to synonymous substitution rate. At least 18 of these genes potentially participate in RNA splicing, DNA repair, glutathione metabolism and the plant–pathogen interaction pathway, which were further enriched in various functional gene categories possibly responsible for environment adaptation in high mountains. Meanwhile, we detected various transcription factors that regulated the material and energy metabolism in *N. incisum* and *N. franchetii*, which probably play vital roles in the tolerance to stress in surroundings. In addition, 60 primer pairs based on orthologous microsatellite-containing sequences between the both *Notopterygium* species were determined. Finally, 17 polymorphic microsatellite markers (SSR) were successfully characterized for the two endangered species. Based on these candidate orthologous and SSR markers, we detected that the adaptive evolution and species divergence of *N. incisum* and *N. franchetii* were significantly associated with the extremely heterogeneous environments and climatic oscillations in high-altitude areas. This work provides important insights into the molecular mechanisms of adaptation to high-altitudes in alpine herbal plants.

## 1. Introduction

Extreme environments provide a natural laboratory for studies on the processes of speciation and adaptive evolution of organisms [1]. In particular, the extremely high-alpine environment is known for its harsh conditions, characterized by severe cold, intense ultraviolet radiation, hypoxia, poor soils, and low CO_2_ pressure [2,3]. Therefore, survival in extreme environments is very challenging for most organisms. Nevertheless, many plant species can thrive in the cold and hypoxic conditions of high-alpine areas [4,5]. Understanding how organisms adapt to these various extreme environments can make a significant contribution to the study of species divergence and evolutionary ecology. Generally, in response to the bioclimatic conditions, plants can generate particular structures and physiological features, and furthermore they can adapt to complex ecological conditions [6]. For example, some alpine plants are able to activate antioxidants, such as ascorbate oxidase (APX), catalase (CAT), and glutathione reductase (GR), proline and abscisicacid, conferring plants with tolerance to the alpine environment [7,8,9].

In recent years, genome/transcriptome sequencing has been proven to be an effective and rapid method for determining adaptive evolution and differential gene expression in high-altitude plants, e.g., *Kobresia pygmaea* [7], *Potentilla saundersiana* [10], *Lamiophlomis rotata* [11] and *Lobelia* [12]. Some studies demonstrated that the alpine plants have various morphological and physiological response strategies to adapt to high-elevation environments. For example, researchers have revealed that some genes are significantly involved in energy metabolism, hypoxia response under positive selection, and rapid adaptation [10,11,12]. Meanwhile, RNA-sequencing (RNA-seq) can also provide important genetic information on the development of molecular markers (e.g., co-dominant microsatellite markers (SSRs) and single-copy nuclear genes) [13]. These genetic markers identified by RNA-sequencing have also been widely applied to study population genetic divergence and species conservation [1,5,13]. However, up to now, few genome/transcriptome-based investigations have been devoted to the molecular mechanisms of high-altitude adaptation and evolution in alpine plants.

*Notopterygium incisum* C. C. Ting ex H. T. Chang and *Notopterygium franchetii* H. de Boissieuas are two important traditional Chinese medicine plants endemic to China. Both species are mainly distributed in the high-alpine meadows and forests (*N. incisum*, 3500–5100m) and sub-alpine shrubs (*N. franchetii*, 1700–4500 m) of Northwest China [14]. They provide excellent models for studying speciation and plant adaptation to extreme environments. In recent decades, wild population resources of the two species has rapidly decreased due to the effects of climatic oscillations and human activities [14]. They are listed as endangered herbal species in the IUCN Red List, and urgent management and conservation are required [15]. Information on population genetic structure and gene diversity are important to formulate effective conservation and management strategies of wild plant resources. However, most previous studies of the two species have mainly focused on their physiological and morphological characteristics [16,17,18], phylogenetic relationships [19,20] and pharmacognosy [21]. Little is known about their population genetics and species divergence information. In addition, the absence of genomic and transcriptomic resources has largely hindered the studies of adaptive evolution of two species to high-altitude areas.

Here, we present large-scale transcriptomes for *N. incisum* and *N. franchetii* that were derived using RNA-seq. Our aims were to: (1) identify candidate genes associated with adaptation to alpine environments through analysis of the functional positively selected genes between two species; (2) increase the limited molecular genetic resources of *Notopterygium* species; and (3) discover transcription factors (TFs) and genus-specific SSR genetic markers for two endangered species.

## 2. Results

### 2.1. Sequencing and De Novo Assembly

**Figure 1:**
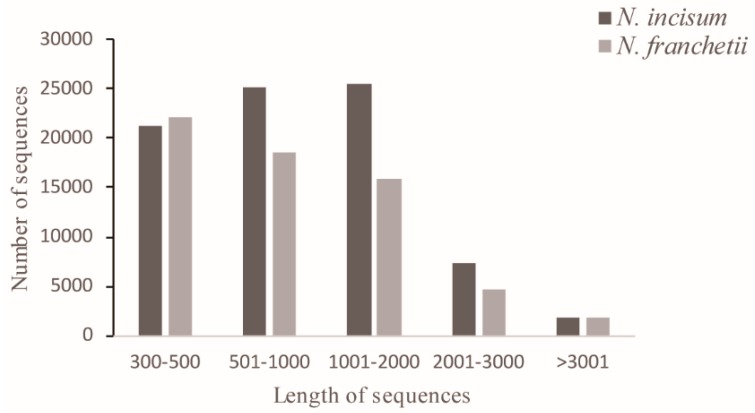
Overview of *Notopterygium incisum* and *Notopterygium franchetii* assemblies and annotations.

In total, 56,759,848 and 61,120,020 sequence reads were generated for *N. incisum* and *N. franchetii*, respectively. After trimming low-quality sequences, we obtained 55,833,880 and 60,052,340 clean reads for the two species, respectively. All of the clean reads were then administered to the Trinity program to assemble transcripts. For *N. incisum*, 81,446 unigenes were obtained with a mean length of 1067 bp and an N50 size of 1435 bp. For *N. franchetii*, 63,153 unigenes were obtained with a mean length of 991 bp and an N50 size of 1405 bp (Table 1, Figure 1).

### 2.2. Functional Annotation

The complete set of unigenes from both *Notopterygium* species was obtained against the NR, GO, Swiss-prot, KOG and KEGG databases. In total, 69,237 (85.0%) sequences from *N. incisum* and 47,774 (75.65%) from *N. franchetii* generated at least one significant alignment with an available sequence in BLASTX searches (Table 2). The top hit species were similar in both *Notopterygium* datasets. The majority of the top hits were genes derived from *Vitis vinifera*, *Sesamum indicum* and *Theobroma cacao* (Figure S1).

**Figure 2:**
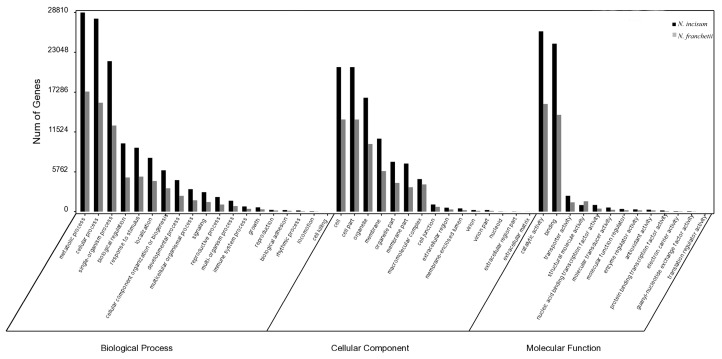
Gene Ontology (GO) distributions of unigenes.

The GO analysis distributions of the unigenes for both herbal species were also highly similar (Figure 2). The GO classification separated the unigenes into 47 functional groups representing biological process (127,779 unigenes from *N. incisum* and 71,679 from *N. franchetii*), cellular component (90,356 unigenes from *N. incisum* and 55,483 from *N. franchetii*) and molecular function (56,434 unigenes from *N. incisum* and 33,848 from *N. franchetii*). Large groups of unigenes from both endangered species (48,515 and 29,474 from *N. incisum* and *N. franchetii*, respectively) were assigned to GO categories. We then used the public KOG database to elucidate the orthologous functions of the unigenes. The distributions of unigene function classification for the two herbal species were also similar (Figure S2).

### 2.3. Putative Orthologs, Substitution Rates, and Species Divergence between N. incisum and N. franchetii

To compare the conservativeness between *N. incisum* and *N. franchetii*, we estimated 56,999 pairs of putative orthologous unigenes between two species applying the reciprocal best hit method with Blastp algorithm (*E*-values < 10^−7^). The remaining protein coding genes that could not be assigned to orthologous groups were considered as species-specific expressed genes. After rigorous filtration, we finally obtained 6375 pairs of single-copy orthologs between the two species by OrthoMCL analysis. These orthologs were used in the subsequent evolutionary analysis of synonymous (Ks) and nonsynonymous (Ka) substitution rates. Finally, a total of 3823 pairs of single copy orthologous genes were identified. Of these orthologs, 381 pairs with a Ka/Ks value > 1 were identified indicating positive selection, and 857 had a Ka/Ks ratio between 0.5 and 1, suggesting weak purifying selection (Figure S3) [22].

**Figure 3:**
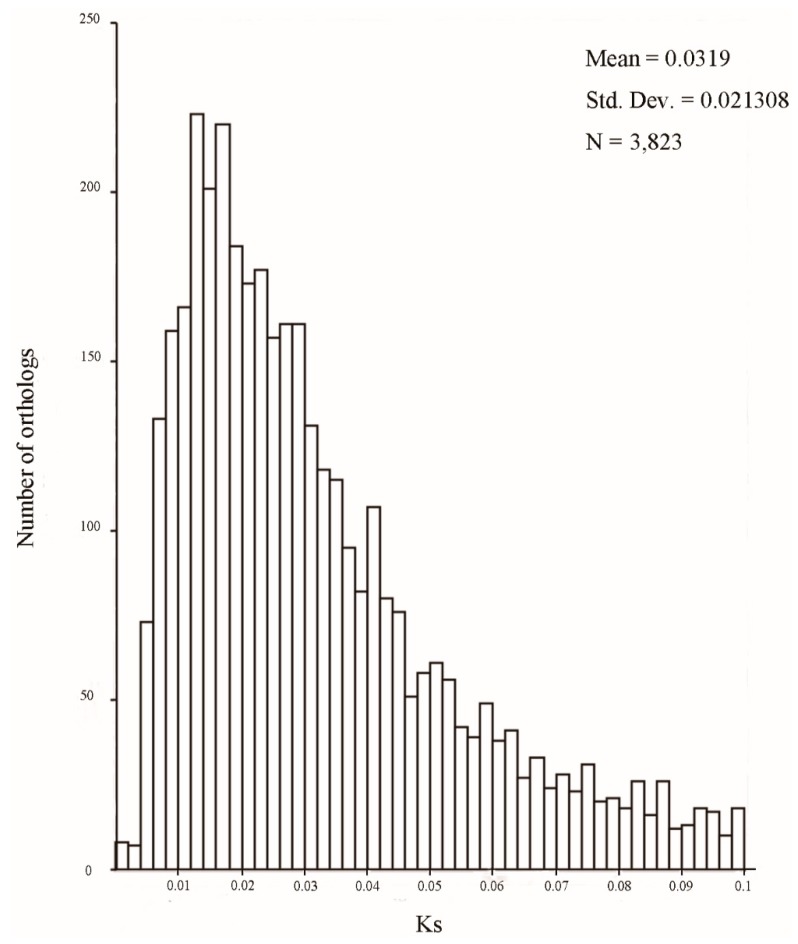
The Ks distribution of orthologs between *Notopterygium incisum* and *Notopterygium franchetii*; their divergence is shown by the peak of Ks at 0.011 ± 0.021.

The divergence times between *N. incisum* and *N. franchetii* can be estimated by utilizing the peak synonymous rates (Ks) of orthologous pairs [13]. In this study, a peak of Ks distribution between two species was observed at 0.011 ± 0.021 (Figure 3). We obtained an approximately estimate of the divergence time (T) between two endangered species according to the simple formula: T = K/2r [23], where r is the synonymous substitution rate, and is considered to be 1.5 × 10^−8^ substitutions/synonymous site/year for the dicots [23]; K is genetic divergence of synonymous substitutions between orthologs. We calculated the period of the species divergence between *N. incisum* and *N. franchetii* to be roughly 1.06 ± 0.71 Mya, which falls between the largest glaciation in the Middle Pleistocene [24,25].

### 2.4. Functions and Metabolite Pathway Analysis under Positive Selection

To detect genes that might be involved in the adaptation to high altitudes, we used 381 pairs of PSGs in orthologous unigenes as the candidate genes, then combined rigorous selection methods to annotate all of the genes based on their functional roles (Table S2 and Figure S4). Firstly, genes derived from significant enrichment analysis included GO and KEGG enrichments, which included putative candidate genes and related metabolic pathways. Secondly, we utilized the functional annotated information for every PSG and pathways to identify the resistance genes reported in previous experimental studies. 

In enrichment analyses, we separated the orthologs into two databases: *N. incisum* and *N. franchetii* with Ka/Ks > 1. In analysis of the GO category with a minimum of five hits, 5 GO-categories annotated to 9 ortholog pairs were detected to be over-represented (Fisher’s exact test, *p*-value < 0.05) in the two *Notopterygium* databases (Table 3). Among these ortholog genes under positive selection with Ka/Ks > 1, most were enriched with functions related to RNA splicing, mismatch repair and acetate metabolic process. In addition, KEGG enrichment analysis revealed that three predicated metabolic pathways were associated with glutathione metabolism, plant–pathogen interaction and ribosome biogenesis in eukaryotes.

**Figure 4:**
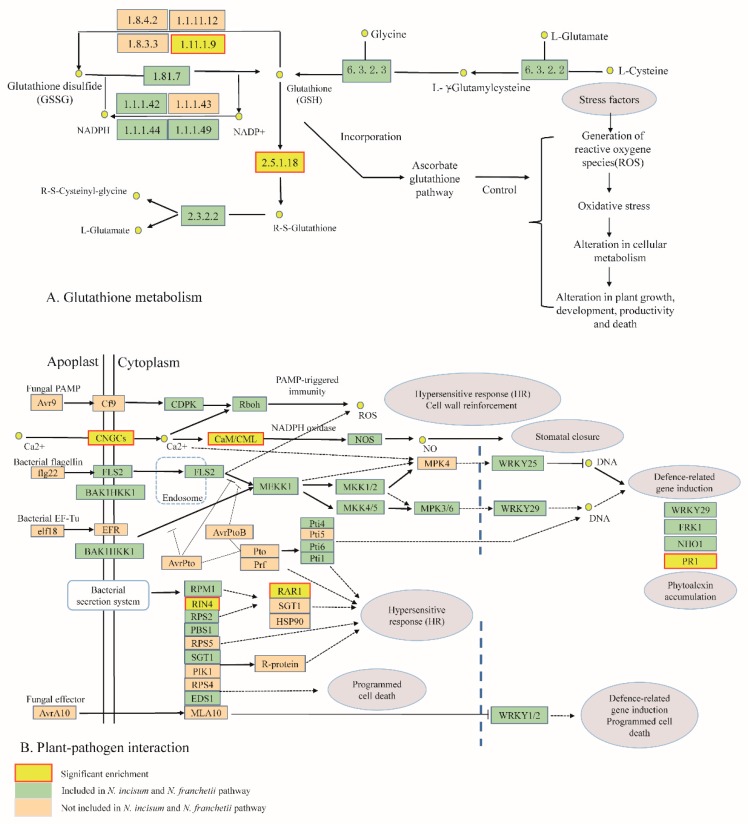
The glutathione-to-glutamate pathway and the plant–pathogen interaction pathway was adapted from the Kyoto Encyclopedia of Genes and Genomes (KEGG) pathway available online (http://www.genome.jp/kegg/pathway.html) [28]. (**A**) Many genes identified by KEGG as encoding the glutathione S-transferase (EC.2.5.1.18) and glutathione peroxidase (EC:1.11.1.9) are enriched in *Notopterygium incisum* and *Notopterygium franchetii*; (**B**) Many genes identified by KEGG as encoding the cyclic nucleotide gated channel (CNGC); CML (CaM/CML), calcium-binding protein, and pathogenesis-related protein 1 (PR1) are enriched in *Notopterygium incisum* and *Notopterygium franchetii*.

Meanwhile, we identified 18 candidate PSGs from Go and KEGG enrichment analysis. These PSGs were mainly involved in RNA-binding protein (RBPs), DNA mismatch repair protein (MSH4), and cysteine and histidine-rich domain-containing protein RAR1, etc., in two *Notopterygium* species (Table 3, Tables S2 and S3). Specifically, most of the genes were involved in resistance genes (GO0008380, GO0032300, GO0031072, KO04620) or enzymes (GO0008083, GO0019783, KO00480, KO03008) in plant metabolite biosynthesis pathways, and were differentially expressed in the two species. However, we noticed that many more unigenes were related to disease resistance (KO04620), antioxidants (KO00480), and cold stress (GO32300, GO0031072, GO0008380), especially antioxidants associated with the glutathione metabolism pathway. As an important antioxidant, glutathione is synthesized from glycine, glutamate and methionine-derived cysteine (Figure 4A). Glutathione is able to recycle with the oxidation/reduction cycle of its reduced (GSH) and oxidized (GSSG) forms [26]. The abundance of glutathione increases when plants have tolerance to oxidative stimuli [27]. Two genes (GP and RSG), encoding the enzymes and regulatory proteins in glutathione biosynthesis, were differentially expressed and highly expressed in high altitude species *N. incisum* (Table S3)*.* Another important metabolic pathway is with respect to the plant–pathogen interactions, here, *N. incisum* and *N. franchetii* showed differential modulation of the expression genes that encoded five proteins included in this pathway (Figure 4B). PAMP-triggered immunity constituted the first line of inducible defense against infectious diseases. Several genes involved in this primary response were linked to cytosolic Ca^2+^ concentrations, including calcium-dependent protein kinase (CDPK), calcium-binding protein CML (CaM/CML) and the cyclic nucleotide gated channel (CNGC), which showed positive selection in both species. Meanwhile, the increase of Ca^2+^ also have regulated the production of ROS and enhanced the tolerance of plants to environment stress. Two pathogen-related genes like PR1, RAR1 were identified, which were upregulated in *N. incisum* compared with *N. franchetii* (Table S3).

### 2.5. Transcription Factor Identification 

**Figure 5:**
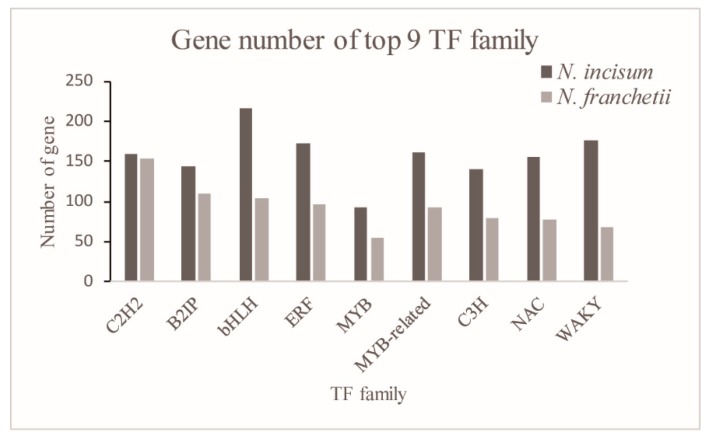
Transcript distribution in various transcription factor (TF) families.

Transcription factors (TFs) played an important role in the plant growth and development [29]. Here, a total of 2696 and 1547 potential TFs were identified by comparing the unigenes of *N. incisum* and *N. franchetii*, respectively. The potential TFs were distributed in 53 families, such as bHLH, MYB, WAKY, ERF, bZIP, and so on (Figure 5). Among these TF gene families, we identified some TFs were under positive selection, and most of them were differentially expressed, which may regulate the downstream target genes involved in stimulus responses in *Notopterygium* (Table 3 and Table S3).

### 2.6. SSR Identification

To assess the quality of the transcriptome assembly and characterize new molecular genetic markers, 6375 pairs of single copy homologous genes were used to develop potential SSRs. A total of 1184/1007 (*N. incisum*/*N. franchetii*) SSR loci primers were identified using the MISA program, of which 155/126 sequences contained more than one SSR repeat (Table 3). The most common repeat type was di-nucleotide, followed by tri-nucleotide, tetra-nucleotide, hexa-nucleotide, and penta-nucleotide (Figure S5). The most abundant repeat motif of di-nucleotide was AT/AT, followed by AC/GT and AG/CT. Among the units of tri-nucleotide, the dominant type was AAG/CTT, followed by ATC/ATG and AGC/CTG (Figure S5).

### 2.7. Genetic Diversity and Divergence of N. incisum and N. franchetii

For evaluating genetic diversity and divergence of *N. incisum* and *N. franchetii*, 45 individuals (Table S1) from nine natural populations were analyzed using 17 polymorphic primers selected from 60 SSR genetic markers (Table S6). Based on these polymorphic primers, the number of alleles (*N*a) per locus for the two species ranged from one to three, with an average value of 1.71. Mean expected heterozygosity (*H*_E_) across all loci in *N. incisum* was 0.201 which was higher than that in *N. franchetii* (*H*_E_ = 0.192). Mean Shannon’s information index (*I*) values were 0.336 and 0.288 for *N. incisum* and *N. franchetii*, respectively (Table S7).

The most likely number of clusters (*K*) of STRUCTURE analysis can be inferred by the peak of Δ*K*. We found that *K* = 2 was the optimal *K* value for SSR (Figure S6), representing two species, respectively. Following 20 independent STRUCTURE runs with *K* = 2, individuals from *N. incisum* were assigned to one cluster with high probability, whereas those samples of *N. franchetii* were assigned to the other cluster with a similarly high probability. When *K* = 3, population E, HA and HH of *N. incisum* exhibits significant genetic divergence and structure (Figure S6).

## 3. Discussion

### 3.1. De Novo Assembly and Functional Annotation

In the present study, the large-scale transcriptomes of two *Notopterygium* species were characterized by de novo sequencing and assembly methods [30]. In total, 55.8 million and 60.1 million clean reads were generated for *N. incisum* and *N. franchetii*, respectively. Also, the unigenes sizes obtained in this study (average length of 1067 bp for *N. incisum* and 991 bp for *N. franchetii*) are longer than those previously published herbal plants using similar technologies [14,31], thus indicating that the transcriptome sequencing data were well assembled for two *Notopterygium* species.

In addition, a substantial part of the unigenes of both species (85.0%/75.65%) could be annotated using four public protein databases and most were involved in plant proteins. Generally, the present study fills an important knowledge gap, and provides relevant transcriptome information regarding the pattern of gene expression and functional categories. Intriguingly, a higher number of unigenes was obtained for two *Notopterygium* species, and the number of annotated unigenes for *N. incisum* (81,446) was greater than that for *N. franchetii* (63,153). This difference suggested the potential to discover novel genes specific to the aim plant, and which might play a key role in species adaptations and evolution [32].

### 3.2. Species Divergence between N. incisum and N. franchetii

Generally, *N. incisum* inhabits the higher mountain areas than *N. franchetii*. They encounter the different environmental conditions and have been shown to have different tolerances during their growth and development [33]. The harsh high-altitude environment imposes strong selective pressures on high alpine plant species. Candidate adaptive genes for high altitudes identified in our study greatly enhance our understanding of the complex genetic architecture, which serves as an important basis for comparative genomic studies of adaptation and species divergence at high elevations.

In addition, peak values of the Ks distribution of orthologs between species often shows species divergence events [34], and this method has been successfully applied in the inference of such divergence events [35,36]. We dated the age of the species divergence between *N. incisum* and *N. franchetii* according to the Ks distribution; it approximately at 1.06 ± 0.71 Mya, which was significantly associated with the rapid uplift of the Qinghai-Tibetan Plateau and the largest glaciation recycles in the Middle Pleistocene [24,25].

### 3.3. Detecting Candidate Genes under Positive Selection

Ka/Ks values are widely used to elucidate protein coding genes under positive selection [37]. We focused on the function of positively selected genes between two *Notopterygium* species (Table S2). These genes associated with GO functional categories clearly indicated the molecular and cellular events involved in *N. incisum* and *N. franchetii* (Figure S4). We identified several interesting candidate genes exhibiting rapid evolution pattern with signs of strong selection (Ka/Ks > 1) in different functional groups that are highly likely associated with the environmental adaptation and stress response. Among them, four genes (ORTHOMCL16216, ORTHOMCL16135, ORTHOMCL16124, ORTHOMCL17922) were found to most likely be associated with the adaptive process to high-elevation environments. Five (ORTHOMCL12869, ORTHOMCL14798, ORTHOMCL15032, ORTHOMCL17505, ORTHOMCL18047) candidate PSGs are involved in links to RBPs, corresponding to the functional group of RNA splicing. Plant genomes encode a variety of RBPs which play important roles in plant growth and stress responses [38]. Serine/arginine-rich (SR) proteins play key roles in precursor mRNA splicing and have been proven to be crucial in plant adaptation to various environments [39]. In addition, one candidate PSG, MSH4, can be related to the mismatch repair (MMR) system. Similarly, MMR proteins play an important role in maintaining genome stability in organisms [40]. Meanwhile, we also have found that the vital gene RAR1 is a component of defense signaling pathways, requiring activation to prevent the spread of infection in the process of plants disease resistance. 

Meanwhile, a series of other defense responses including glutathione metabolism and plant–pathogen interaction was identified (Figure 4A), which was of vital importance in the process of adaptive evolution and species divergence of plants [41]. We also detected a large number of expressed genes encoding various enzymes associated with glutathione metabolism, indicating that glutathione may play a key role in the adaptation to the high mountain environments for the two endangered species (Table S4). 

In addition, most of the expressed genes encoding various enzymes involved in plant–pathogen interaction metabolism were also predicted (Table S5). The PAMP-triggered immunity pathway (PTI) was activated (Figure 4B), particularly by the up-regulation of the expression level of calcium-dependent protein kinase CDPK. Therefore, the activation of the PTI inhibits pathogen of *N. incisum* and *N. franchetii*, which made these two species have more widely adaptation to the harsh environments [42,43].

Furthermore, some important transcription factors were determined in two high altitude species. Generally, TFs can regulate the downstream genes involved in responses to environment stress, e.g., MYB, NAC, WRKY and ERF etc. [29,44]. These TFs have been proven to be significantly associated with various environment stresses in many plants [36,45,46]. Firstly, NAC proteins constituted one of the largest families of plant transcription factors [47]. Genes from this family participated in a large number of biological processes including developmental programs, defense, and environment stress responses [48,49]. In this study, two unigenes (ORTHOMCL17007 and ORTHOMCL20774) were identified in NAC gene networks. Secondly, the WRKY transcription factor may be associated with plant abiotic stresses such as cold and drought conditions [50,51]. Two unigenes (ORTHOMCL11146 and ORTHOMCL13242) were annotated as WRKY transcription factors which were under positive selection in two *Notopterygium* species. Thirdly, we found two genes (ORTHOMCL16173 and ORTHOMCL17851) belonging to the MYB family TFs, which were induced in various environment stress responses and tolerances [52,53]. In addition, basic helix-loop-helix (bHLH) TFs comprised a large TF family and acted as crucial regulators in response to stress in plants [54,55] were identified. Another two of the TFs, BkERF2.1, DcERF1, are both associated with enhanced disease resistance. BkERFs contained an ERF/AP2 DNA binding domain, which mediated the expression of defense-related genes in plants [56]. DcERF1, belonging to the ethylene-responsive element-binding factor (ERF) family (related to plant defense [57,58], regulation of secondary metabolism and the flower senescence) was also found in this study.

### 3.4. Polymorphic SSR Markers and Population Structure

Genetic markers based on transcriptome sequences are effective for studying population structure, diversity and species divergence [59]. To validate the determined SSR markers, we amplified the predicted SSR primers via PCR reaction. Of the 60 pair primers that were selected for PCR validation, 28 (46.67%) produced clear DNA bands. This PCR success rate was higher than that reported for some herbal plants such as alfalfa (30%) [60] and tree peony (47.30%) [61]. Among the working primers, we excluded 11 primers which showed polymorphism in one species but could not amplify clear bands in another, and then 17 polymorphic primer pairs were selected as genus-specific SSR markers to evaluate genetic diversity and divergence of *N. incisum* and *N. franchetii*. Genetic diversity analyses suggested that most of these primers showed higher polymorphism in *N. incisum* than in *N. franchetii*. The mean expected heterozygosity (*H*_E_) across all loci in *N. incisum* was 0.201 and was higher than that in *N. franchetii* (*H*_E_ = 0.192). This was probably caused by the much more limited distributional range and natural populations of *N. incisum* than *N. franchetii*. Meanwhile, according to the STRUCTURE cluster analysis, we found that all populations were clustered into two groups (Figure S6) and these showed a clear demarcation between accessions from two species (Figure S6). The results implied that there may exist high genetic divergence or/and barriers between *N. incisum* and *N. franchetii*. In short, our current study indicated that the SSR primers from two species transcriptomes are reliable and could be used to study the population genetics and species divergence of *Notopterygium* in the future.

## 4. Materials and Methods

### 4.1. Plant Material

The fresh tissues of *N. incisum* and *N. franchetii* used in this study were collected in June 2013, from the Taibai Mountain (33°59′57.322″ N, 107°48′25.527″ E, 3500 m), Shaanxi province, and Gansu province (33°29′09.655″ N, 104°31′30.857″ E, 1800 m), in the western region of China. To obtain as many expressed genes as possible, three different tissues were sampled for each species, including leaves, stems and flowers. We immediately froze tissue samples in liquid nitrogen and stored them at −80 °C in a cryogenic refrigerator until RNA extraction. Fresh young leaves of 45 individuals of both *Notopterygium* species were also collected from Sichuan, Gansu, and Shaanxi, China for SSR development and validation (Table S1). Samples were dried instantly using silica gel for DNA extraction. 

### 4.2. RNA Preparation, Illumina Paired-End Sequencing

We isolated the total RNA of two *Notopterygium* species using the RNeasy Kit (Qiagen, Hilden, Germany) [13]. A mixed sample with equal volumes (RNAs of leaves, stems and flowers) was prepared as a single pooled RNA sample for each species. cDNA preparation and RNA-Seq were carried out using these pooled samples. Double-stranded cDNA was synthesized and sequencing adaptors were ligated according to the manufacturer’s instructions (Illumina, San Diego, CA, USA). The ligated products were then purified with AMPureXP beads and amplified for the construction of cDNA libraries [62]. The libraries were sequenced using the Illumina HiSeq^TM^ 2000 (Novogene, Tianjin, China) after completion of cDNA libraries. The raw transcriptome datasets of *N. incisum* and *N. franchetii* were deposited in the NCBI Sequence Read Archive (accession numbers SRR5573673 and SRR5659683, respectively). 

### 4.3. Transcriptome Assembly and Function Annotation

We obtained clean reads by filtering the adaptor sequences, low-quality regions (bQ20) and sequences shorter than 50 bp [63]. The program Trinity was employed for de novo assembly with all clean reads (http://trinityrnaseq.sourceforge.net/) [64]. BLASTx was used to align unigene sequences to the protein databases NCBI non-redundant (NR), the Swiss-Prot protein database (Swiss-Prot, in UniProt), the Kyoto Encyclopedia of Genes and Genomes (KEGG, www.genome.jp/kegg/) [28], and the Clusters of enKaryotic Orthologous Groups of proteins (KOG, http://www.ncbi.nlm.nih.gov/KOG/) [65].The highest similarity protein sequences with E-values < 10^−5^ were selected and then analyzed. According to NR annotation, Gene Ontology (GO) annotation and classification of all unigenes was acquired by the Blast2Go program [66] and WEGO software [67], which comprehensively describe the attributes of genes and gene products in species [68].

### 4.4. Ortholog Identification and Sequence Alignment

We used the BLASTx and ESTScan software to predicate the coding sequences (CDSs and protein sequences) [69]. The latter is aimed at unigenes that do not align with the previously mentioned protein databases. Then, the predicted CDSs allowed us to evaluate orthologous groups between *N. incisum* and *N. franchetii*. Blastp search of the predicted CDS regions of two species transcriptomes with an *E*-values < 10^−7^was carried out. For such comparisons, the purpose of the alignment is to appraise gene families and potential orthologs [32]. These analyses were performed with the program OrthoMCL [70].

### 4.5. Substitution Rate Estimation and Selection Analyses

Putative single-copy orthologs were assessed by clustering protein sequences between *N. incisum* and *N. franchetii* (OrthoMCL). The nonsynonymous (Ka), synonymous (Ks), and Ka/Ks values were computed using the software KaKs_Calculator (http://code.google.com/p/kaks-calculator/wiki/KaKs_Calculatoref) [71]. We used the Fisher’s exact test to validate the efficiency of the Ka and Ks values for each single ortholog. We excluded candidate orthologs with a synonymous (Ks) substitution value >0.1, as these may be paralogs [13,22]. Rigorous criteria were used to identify the positive selection genes (PSGs): PSGs were required to have a Ka/Ks value higher than 1 [5]. To speculate on the putative functions of unigenes specifically over-expressed in orthologous groups, GO and KEGG pathways enrichment analyses were performed by comparing homologous genes between two species. Multiple testing was implemented by using the false discovery rate [72], with a cut-off of 0.05 to determine significant over expression.

### 4.6. Transcription Factor Mining

In order to identify some positively selected genes and detect the adaption mechanisms in the two high-alpine herbal species, we analyzed the transcription factors represented in *N. incisum* and *N. franchetii* transcriptomes; all assembled and annotated unigenes were aligned to the plant transcription factor database (PlantTFdb; http://plntfdb.bio.uni-potsdam.de/v3.0/downloads.php) with application of the program HMMER [73].

### 4.7. Microsatellite Marker Detection 

We used the program MISA (http://pgrc.ipk-gatersleben.de/misa/) [74] to mine the microsatellite markers of *N. incisum* and *N. franchetii*. The criteria of SSR search were as follows: di-, tri-, tetra-, penta- and hexa-nucleotide motifs with at least a 6, 5, 4, 4 and 4 repeats, respectively [75]. The maximum size of interruption allowed between two different SSRs in a compound sequence was 100 bp. SSR primer pairs were designed using the software Primer Premier 6.0 (PremierBiosoft International, Palo Alto, CA, USA). A random selection of tetra- to penta-nucleotide microsatellites were used to design locus-specific primers for two species [76,77].

### 4.8. DNA Isolation and Primer Selection 

We isolated genomic DNA of dried leaves using the cetyltrimethylammonium bromide method [78]. The quality of DNA was determined by 1% agarose gel electrophoresis. Sixty primer pairs were detected with 45 samples of *N. incisum* and *N. franchetii*. Polymerase chain reaction (PCR) amplifications were administered with a 10 μL reactive volume containing 1 μL of DNA template (10–40 ng/μL), 5 μL of 2X Taq mix, 0.4 μL of the forward primer (1 mM), 1.6 μL of the reverse primer (1 mM), and 1.4 μL of ddH_2_O. PCR amplification conditions consisted of an initial denaturation step at 95 °C for 5 min, followed by 32 cycles of 95 °C for 30 s, 53–57 °C for 90 s, and 72 °C for 60 s; and a final elongation step of 7 min at 72 °C. All loci were run separately in 10% non-denaturing polyacrylamide gel and then visualized by silver staining protocols. The band size was determined using the program Quantity One (Bio-Rad Laboratories, Hercules, CA, USA) with PBR322 DNA/MspI as the molecular size standard.

### 4.9. Microsatellite DNA Analysis

A set of statistical tests on SSRs, including allelic richness (*N*a), Shannon’s information index (*I*), and observed and expected heterozygosity (*H*o and *H*_E_, respectively), were performed via the software GenALEx version 6.5 (Canberra, Australia) [79]. Genetic structure of all populations was estimated by clustering method based on a Bayesian model with the software package STRUCTURE [80]. The data set without prior population information was analyzed using an admixture model and independent allelic frequencies [81]. A total of 20 independent simulations were run for *K* from 1 to 6 with 2 × 10^5^ burn-in steps followed by 5 × 10^5^ MCMC steps. The program STRUCTURE HARVESTR (Santa Cruz, CA, USA) was employed to evaluate the most likely number (*K*) of genetic clusters using the delta *K* criterion [82]; the inferred clusters were drawn as colored box-plots using the bio-software DISTRUCT (Los Angeles, CA, USA) [83].

## Figures and Tables

**Table 1:** Summary of transcriptome data for *Notopterygium incisum* and *Notopterygium franchetii*.

	*N. incisum*	*N. franchetii*
Sequencing data		
Total number of reads	55,833,880	60,052,340
Total length of reads (bp)	6,979,235,000	9,007,851,000
GC (%)	40.57	43.91
Q20 (%)	97.09	97.31
Final assembly		
Total number of unigenes	81,446	63,153
Total length of unigenes	86,924,837	62,615,693
N50 length of assembly	1435	1405
Mean of length of assembly	1067	991

**Table 2:** Functional annotation of unigenes for *Notopterygium incisum* and *Notopterygium franchetii*.

	Database	*N. incisum*		*N. franchetii*	
		Number	Percentage (%)	Number	Percentage (%)
Annotated	NR	69,141	84.89	45,267	71.68
	Swiss-Prot	53,805	66.06	39,518	62.58
	KOG	45,705	56.12	33,217	52.6
	KEGG	31,488	38.66	23,288	36.88
	GO	48,515	59.57	29,474	46.67
	Total	69,237	85	47,774	75.65
Unannotated		12,209	14.99	15,379	24.35
Total		81,446	100	63,153	100

KOG: enKaryotic Orthologous Groups of proteins; KEGG: Kyoto Encyclopedia of Genes and Genomes; NR: non-redundant; GO: Gene Ontology.

**Table 3:** Putative genes involved in stress tolerance in orthologous unigenes.

Orthologous Unigenes	GO Terms	BLASTx to NR Database	Ka/Ks
ORTHOMCL12869	RNA splicing	serine/arginine-rich SC35	1.45
ORTHOMCL14798	RNA splicing	RNA-binding protein with multiple splicing	1.04
ORTHOMCL15032	RNA splicing	Serine/arginine-rich splicing factor 12	1.66
ORTHOMCL17505	RNA splicing	U1 small nuclear ribonucleoprotein （U1 snRNP）	1.22
ORTHOMCL18047	RNA splicing	tRNA 2′-phosphotransferase 1	2.93
ORTHOMCL16216	mismatch repair	DNA mismatch repair protein MSH4	1.15
ORTHOMCL16135	acetate metabolic process	acetyl-coenzyme A synthetase	1.98
ORTHOMCL16124	heat shock protein binding	cysteine and histidine-rich domain-containing protein RAR1	1.2
ORTHOMCL17922	catalytic-type peptidase activity	NEDD8-specific protease 1	2.4
	**KEGG pathway**		
ORTHOMCL11569	Plant–pathogen interaction	RPM1 interacting protein 4 transcript 2	1.3
ORTHOMCL16124	Plant–pathogen interaction	cysteine and histidine-rich domain-containing protein RAR1	1.2
ORTHOMCL13297	Plant–pathogen interaction	probable calcium-binding protein CML45	1.61
ORTHOMCL17232	Plant–pathogen interaction	pathogenesis-related protein PR-1 type	4.32
ORTHOMCL18122	Plant–pathogen interaction	probable cyclic nucleotide-gated ion channel 17	2.93
ORTHOMCL17558	Glutathione metabolism	glutathione peroxidase	1.21
ORTHOMCL17864	Glutathione metabolism	glutathione S-transferase	1.54
ORTHOMCL15235	Ribosome biogenesis in eukaryotes	nucleolar protein 56	5.42
ORTHOMCL11943	Ribosome biogenesis in eukaryotes	probable mediator of RNA polymerase II transcription subunit 36b	1.09
ORTHOMCL20729	Ribosome biogenesis in eukaryotes	nucleolar protein 56-like	1.37
	**Transcription factors**		
ORTHOMCL11146	WRKY	WRKY transcription factor 48	1.02
ORTHOMCL13242	WRKY	WRKY transcription factor 22	2.35
ORTHOMCL17007	NAC	NAC transcription factor	1.05
ORTHOMCL20774	NAC	NAC domain-containing protein 21/22	1.16
ORTHOMCL16173	MYB	MYB-like protein X	3.22
ORTHOMCL17851	MYB	transcription factor MYB48 isoform X1	1.74
ORTHOMCL11581	bHLH	transcription factor bHLH61 isoform 1	1.05
ORTHOMCL14612	bHLH	transcription factor bHLH81	1.16
ORTHOMCL12079	bHLH	transcription factor bHLH100	1.37
ORTHOMCL11075	bHLH	transcription factor bHLH130	2.61
ORTHOMCL18712	ERF	transcription factor DcERF1	2.29
ORTHOMCL11417	ERF	ethylene response factor 2.1	1.14

## Supplementary Materials

Supplementary materials are available online.

## References

[1] Qiao Q., Wang Q., Han X., Guan Y., Sun H., Zhong Y., Huang J., Zhang T. (2016). Transcriptome sequencing of *Crucihimalaya himalaica* (Brassicaceae) reveals how Arabidopsis close relative adapt to the Qinghai-Tibet Plateau. Sci. Rep..

[2] Wiener G., Han J.L., Long R.J. (2003). The Yak.

[3] Xin G.S., Long R.J., Guo X.S., Irvine J., Ding L.M., Ding L.L., Shang Z.H. (2011). Blood mineral status of grazing Tibetan sheep in Northeast of the Qinghai-Tibetan Plateau. Livest. Sci..

[4] Storz J.F., Scott G.R., Cheviron Z.A. (2010). Phenotypic plasticity and genetic adaptation to high-altitude hypoxia in vertebrates. J. Exp. Biol..

[5] Yang Y., Wang L., Han J., Tang X., Ma M., Wang K., Zhang X., Ren Q., Chen Q., Qiu Q. (2015). Comparative transcriptomic analysis revealed adaptation mechanism of *Phrynocephalus erythrurus*, the highest altitude Lizard living in the Qinghai-Tibet Plateau. BMC Evol. Boil..

[6] Guo N., Gao J., He Y., Guo Y. (2016). Compositae plants differed in leaf cuticular waxes between high and low altitudes. Chem. Biodivers..

[7] Li X., Yang Y., Ma L., Sun X., Yang S., Kong X., Hu X., Yang Y. (2014). Comparative proteomics analyses of *Kobresia pygmaea* adaptation to environment along an elevational gradient on the Central Tibetan Plateau. PLoS ONE.

[8] Foyer C.H., Noctor G. (2005). Redox homeostasis and antioxidant signaling: A metabolic interface between stress perception and physiological responses. Plant Cell.

[9] Vinocur B., Altman A. (2005). Recent advances in engineering plant tolerance to abiotic stress: Achievements and limitations. Curr. Opin. Biotechnol..

[10] Ma L., Sun X., Kong X., Galvan J.V., Li X., Yang S., Yang Y., Yang Y., Hu X. (2015). Physiological, biochemical and proteomics analysis reveals the adaptation strategies of the alpine plant *Potentilla saundersiana* at altitude gradient of the Northwestern Tibetan Plateau. J. Proteomics.

[11] Ma L., Yang L., Zhao J., Wei J., Kong X., Wang C., Zhang X., Yang Y., Hu X. (2015). Comparative proteomic analysis reveals the role of hydrogen sulfide in the adaptation of the alpine plant *Lamiophlomis rotata* to altitude gradient in the Northern Tibetan Plateau. Planta.

[12] Zhao S.Y., Chen L.Y., Muchuku J.K., Hu G.W., Wang Q.F. (2016). Genetic adaptation of giant lobelias (*Lobelia aberdarica* and *Lobelia telekii*) to different altitudes in East African mountains. Front. Plant Sci..

[13] Zhang L., Yan H.F., Wu W., Yu H., Ge X.J. (2013). Comparative transcriptome analysis and marker development of two closely related Primrose species (*Primula poissonii* and *Primula wilsonii*). BMC Genom..

[14] Zhou G., Yang L., Li C., Xu W., Chen G. (2010). Genetic diversity in endangered *Notopterygiumforbesii* Boissieu based on intraspecies sequence variation of chloroplast DNA and implications for conservation. Biochem. Syst. Ecol..

[15] Wu Z.Y., Raven P.H. (2005). Apiaceae through Ericaceae, In Flora of China.

[16] Wang Y.P., Pu F., Wang P., He X.J. (1996). Studies on the systematics of the Chinese endemic genus Notopterygium. Acta Bot. Yunnanica.

[17] She M., Pu F. (1996). A new species of Notopterygium de Boiss. from China. J. Plant Resour. Environ..

[18] Jiang S.Y., Sun H., Huang X.J., Zhou Y., Ma X.J., Yang Z.R. (2005). Environmental pedology of *Notopterygium incisum* and *N. forbesii*. Chin. Tradit. Herb. Drugs.

[19] Pu F., Wang P., Zheng Z., Wang Y.P. (1999). A reclassification of *Notopterygium boissieu* (Umbelliferae). Acta Phytotaxon. Sin..

[20] Yang J., Yue M., Niu C., Ma X.F., Li Z.H. (2017). Comparative Analysis of the Complete Chloroplast Genome of Four Endangered Herbals of Notopterygium. Genes.

[21] Yang X.W., Zhang P., Tao H.Y., Jiang S.Y., Zhou Y. (2006). GC-MS analysis of essential oil constituents from rhizome and root of *Notopterygium forbesii*. J. Chin. Pharm. Sci..

[22] Zhou T., Chen C., Wei Y., Chang Y.X., Bai G.Q., Li Z.H., Kanwal N., Zhao G.F. (2016). Comparative Transcriptome and Chloroplast Genome Analyses of Two Related *Dipteronia* Species. Front. Plant Sci..

[23] Koch M.A., Haubold B., Mitchell-Olds T. (2000). Comparative evolutionary analysis of chalcone synthase and alcohol dehydrogenase loci in *Arabidopsis*, *Arabis*, and related genera (Brassicaceae). Mol. Boil. Evol..

[24] Zheng B., Xu Q., Shen Y. (2002). The relationship between climate change and Quaternary glacial cycles on the Qinghai-Tibetan Plateau: Review and speculation. Quat. Int..

[25] Sun Y., Li L., Li L., Zou J., Liu J. (2015). Distributional dynamics and interspecific gene flow in *Picea likiangensis* and *P. wilsonii* triggered by climate change on the Qinghai-Tibet Plateau. J. Biogeogr..

[26] Noctor G., Mhamdi A., Chaouch S., Han Y., Neukermans J., Marquez Garcia B., Queval G., Foyer C.H. (2012). Glutathione in plants: An integrated overview. Plant Cell Environ..

[27] Liu X.M., Zhang S.Z., Whitworth R.J., Stuart J.J., Chen M.S. (2015). Unbalanced activation of glutathione metabolic pathways suggests potential involvement in plant defense against the gall midge Mayetiola destructor in wheat. Sci. Rep..

[28] Ogata H., Goto S., Sato K., Fujibuchi W., Bono H., Kanehisa M. (1999). Kegg: Kyoto encyclopedia of genes and genomes. Nucleic Acids Res..

[29] Bonthala V.S., Mayes K., Moreton J., Blythe M., Wright V., May S.T., Massawe F., Mayes S., Twycross J. (2016). Identification of gene modules associated with low temperatures response in Bambara groundnut by network-based analysis. PLoS ONE.

[30] Birol I., Behsaz B., Hammond S.A., Kucuk E., Veldhoen N., Helbing C.C. (2015). De novo transcriptome assemblies of *Rana* (Lithobates) *catesbeiana* and *Xenopus laevis* tadpole livers for comparative genomics without reference genomes. PLoS ONE.

[31] Zhan X., Yang L., Wang D., Zhu J.K., Lang Z.B. (2016). De novo assembly and analysis of the transcriptome of *Ocimumamericanum* var. *pilosum* under cold stress. BMC Genom..

[32] Rispe C., Fabrice L., Daciana P., Anthony B., Sylvie H., Gaël L.T., Denis T., Julie J., François D. (2016). De novo transcriptome assembly of the grapevine phylloxera allows identification of genes differentially expressed between leaf-and root-feeding forms. BMC Genom..

[33] Zhang J., Xie P., Lascoux M., Meagher T.R., Liu J. (2013). Rapidly evolving genes and stress adaptation of two desert poplars, *Populuseu phratica* and *P. pruinosa*. PLoS ONE.

[34] Wang Y., Hey J. (2010). Estimating divergence parameters with small samples from a large number of loci. Genetics.

[35] Lynch M., Conery J.S. (2000). The evolutionary fate and consequences of duplicate genes. Science.

[36] Blanc G., Wolfe K.H. (2004). Widespread paleopolyploidy in model plant species inferred from age distributions of duplicate genes. Plant Cell.

[37] Hurst L.D. (2002). The Ka/Ks ratio: Diagnosing the form of sequence evolution. Trends Genet..

[38] Lee K., Kang H. (2016). Emerging roles of RNA-binding proteins in plant growth, development, and stress responses. Mol. Cells.

[39] Califice S., Baurain D., Hanikenne M., Motte P.A. (2012). Single Ancient Origin for Prototypical Serine/Arginine-Rich Splicing Factors1. Plant Physiol..

[40] Tuteja R., Tuteja N. (2013). Analysis of DNA repair helicase UvrD from Arabidopsis thaliana and Oryza sativa. Plant Physiol. Biochem..

[41] Ghanta S., Chattopadhyay S. (2011). Glutathione as a signaling molecule: Another challenge to pathogens. Plant Signal Behav..

[42] Jones J.D., Dangl J.L. (2006). The plant immune system. Nature.

[43] Pociecha E., Plazek A., Janowiak F., Waligorski P., Zwierzykowski Z. (2009). Changes in abscisic acid, salicylic acid and phenylpropanoid concentrations during cold acclimation of androgenic forms of Festulolium (*Festuca pratensis*×*Lolium multiflorum*) in relation to resistance to pink snow mould (Microdochium nivale). Plant Breed..

[44] He W., Zhuang H., Fu Y., Guo L., Guo B., Guo L., Zhang Y., Wei Y. (2015). De novo transcriptome assembly of a Chinese locoweed (*Oxytropis ochrocephala*) species provides insights into genes associated with drought, salinity, and cold tolerance. Front. Plant Sci..

[45] Bhardwaj A.R., Joshi G., Kukreja B., Malik V., Arora P., Pandey R., Shukla R.N., Bankar K.G., Katiyar-Agarwal S., Goel S. (2015). Global insights into high temperature and drought stress regulated genes by RNA-Seq in economically important oilseed crop Brassica juncea. BMC Plant Biol..

[46] Zhu M.K., Chen G., Zhang J.L., Zhang Y.J., Xie X.L., Zhao Z.P., Pan Y., Hu Z.L. (2014). The abiotic stress-responsive NAC-type transcription factor SlNAC4 regulates salt and drought tolerance and stress-related genes in tomato (*Solanum lycopersicum*). Plant Cell Rep..

[47] Puranik S., Sahu P.P., Srivastava P.S., Prasad M. (2012). NAC proteins: Regulation and role in stress tolerance. Trends Plant Sci..

[48] Olsen A.N., Ernst H.A., Leggio L.L., Skriver K. (2005). NAC transcription factors: Structurally distinct, functionally diverse. Trends Plant Sci..

[49] Hegedus D., Yu M., Baldwin D., Gruber M., Sharpe A., Parkin I., Whitwill S., Lydiate D. (2003). Molecular characterization of Brassicanapus NAC domain transcriptional activators induced in response to biotic and abiotic stress. Plant Mol. Boil..

[50] Rushton P.J., Somssich I.E., Ringler P., Shen Q.J. (2010). WRKY transcription factors. Trends Plant Sci..

[51] Tripathi P., Rabara R.C., Rushton P.J. (2014). A systems biology perspective on the role of WRKY transcription factors in drought responses in plants. Planta.

[52] Zhen W., Jun T., Rong H., Peng W., Hou X.L., Song X.M., Xiong A.S. (2015). Genome-wide analysis of the R2R3-MYB transcription factor genes in Chinese cabbage (*Brassica rapa* ssp. *pekinensis*) reveals their stress and hormone responsive patterns. BMC Genom..

[53] Muthamilarasan M., Khandelwal R., Yadav C.B., Bonthala V.S., Khan Y., Prasad M. (2014). Identification and molecular characterization of MYB transcription factor superfamily in C4 model plant foxtail millet (*Setariaitalica* L.). PLoS ONE.

[54] Tang Q., Huang W., Guan J. (2015). Transcriptomic analysis provides insight into high-altitude acclimation in domestic goats. Gene.

[55] Kavas M., Baloğlu M.C., Atabay E.S., Ziplar U.T., Daşgan H.Y., Ünver T. (2016). Genome-wide characterization and expression analysis of common bean bHLH transcription factors in response to excess salt concentration. Mol. Genet. Genom..

[56] Liu W.Y., Chiou S.J., Ko C.Y., Lin T.Y. (2011). Functional characterization of three ethylene response factor genes from *Bupleurumkaoi* indicates that BkERFs mediate resistance to Botrytis cinerea. J. Plant Physiol..

[57] Kimura S., Chikagawa Y., Kato M., Maeda K., Ozeki Y. (2008). Upregulation of the promoter activity of the carrot (*Daucuscarota*) phenylalanine ammonia-lyase gene (DcPAL3) is caused by new members of the transcriptional regulatory proteins, DcERF1 and DcERF2, which bind to the GCC-box homolog and act as an activator to the DcPAL3 promoter. J. Plant Res..

[58] Sun Y.Z., Niu Y.Y., Li Y., Zhu Y.J., Luo H.M., Chen S.L. (2011). Cloning and bioinformatic analysis of PqERF1 gene in *Panax quinquefolius*. Acta Pharm. Sin..

[59] Chen W., Liu Y.X., Jiang G.F. (2015). De novo assembly and characterization of the testis transcriptome and development of EST-SSR markers in the cockroach *Periplaneta americana*. Sci. Rep..

[60] Wang Z., Yan H.W., Fu X.N., Li X.H., Gao H.W. (2013). Development of simple sequence repeat markers and diversity analysis inalfalfa (*Medicago sativa* L.). Mol. Biol. Rep..

[61] Wu J., Cai C.F., Cheng F.Y., Cui H.L., Zhou H. (2014). Characterisation and development of EST-SSR markers in tree peony using transcriptome sequences. Mol. Breed..

[62] Li Y.D., Hui M., Cui Z., Liu Y., Song C.W., Shi G.H. (2015). Comparative transcriptomic analysis provides insights into the molecular basis of the metamorphosis and nutrition metabolism change from zoeae to megalopae in *Eriocheirsinensis*. Genom. Proteom..

[63] Cox M.P., Peterson D.A., Biggs P.J. (2010). Solexa QA: At-a-glance quality assessment of Illumina second-generation sequencing data. BMC Bioinform..

[64] Grabherr M.G., Haas B.J., Yassour M., Levin J.Z., Thompson D.A., Amit I., Adiconis X., Fan L., Raychowdhury R., Zeng Q.D. (2011). Full-length transcriptome assembly from RNA-Seq data without a reference genome. Nat. Biotechnol..

[65] Koonin E.V., Fedorova N.D., Jackson. J.D., Jacobs A.R., Krylov D.M., Makarova K.S., Mazumder R., Mekhedov S.L., Nikolskaya A.N., Rao B.S. (2004). A comprehensive evolutionary classification of proteins encoded in complete eukaryotic genomes. Genom. Biol..

[66] Conesa A., Gotz S., Garcia-Gomez J.M., Terol J., Talon M., Robles M. (2005). Blast2GO: A universal tool for annotation, visualization and analysis in functional genomics research. Bioinformatics.

[67] Ye J., Fang L., Zheng H., Zhang Y., Chen J., Zhang Z., Wang J., Li S., Li R., Bolund L. (2006). WEGO: A web tool for plotting GO annotations. Nucleic Acids Res..

[68] Du F., Wu Y., Zhang L., Li X.W., Zhao X.Y., Wang W.H., Gao Z.S., Xia Y.P. (2015). De novo assembled transcriptome analysis and SSR marker development of a mixture of six tissues from Lilium oriental hybrid ‘Sorbonne’. Plant Mol. Biol. Rep..

[69] Iseli C., Jongeneel C.V., Bucher P. (1999). ESTScan: A program for detecting, evaluating, and reconstructing potential coding regions in EST sequences. ISMB.

[70] Li L., Stoeckert C.J., Roos D.S. (2003). OrthoMCL: Identification of ortholog groups for eukaryotic genomes. Genom. Res..

[71] Vatansever R., Koc I., Ozyigit I.I., Sen U., Uras M.E., Anjum N.A., Eduarda P., Filiz E. (2016). Genome-wide identification and expression analysis of sulfate transporter (SULTR) genes in potato (*Solanum tuberosum* L.). Planta.

[72] Castresana J. (2000). Selection of conserved blocks from multiple alignments for their use in phylogenetic analysis. Mol. Biol. Evol..

[73] Kalra S., Puniya B.L., Kulshreshtha D., Kumar S., Kaur J., Ramachandran S. (2013). De novo transcriptome sequencing reveals important molecular networks and metabolic pathways of the plant, *Chlorophytum borivilianum*. PLoS ONE.

[74] Thiel T., Michalek W., Varshney R., Graner A. (2003). Exploiting EST databases for the development and characterization of gene-derived SSR-markers in barley (*Hordeum vulgare* L.). Theor. Appl. Genet..

[75] Zhang J.P., Wu Y., Li D.Q., Wang G.Q., Li X., Xia Y.P. (2015). Transcriptomic analysis of the underground renewal buds during dormancy transition and release in ‘Hangbaishao’ peony (*Paeonia lactiflora*). PLoS ONE.

[76] Jiang B., Xie D., Liu W., Peng Q., He X. (2013). De novo assembly and characterization of the transcriptome, and development of SSR markers in wax gourd (*Benicasa hispida*). PLoS ONE.

[77] Scott M.W., Hoffman J.R., Hewitt T.L., Beasley R.R., Lance S.L., Jones K.L., Morris T.J., Zanatta D.T. (2016). Development and characterization of 29 microsatellite markers for *Ligumianasuta* (Bivalvia: Unionidae) using an Illumina sequencing approach. Biochem. Syst. Ecol..

[78] Doyle J.J. (1987). A rapid DNA isolation procedure for small quantities of fresh leaf tissue. Phytochem. Bull..

[79] Peakall R., Smouse P.E. (2012). GenAlEx 6.5: Genetic analysis in Excel. Population genetic software for teaching and research-an update. Bioinformatics.

[80] Pritchard J.K., Stephens M., Donnelly P. (2000). Inference of population structure using multilocus genotype data. Genetics.

[81] Gao T., Han Z., Zhang X., Luo J., Yanagimoto T., Zhang H. (2016). Population genetic differentiation of the black rockfish Sebastes schlegelii revealed by microsatellites. Biochem. Syst. Ecol..

[82] Earl D.A., von Holdt B.M. (2012). Structure Harvester: A website and program for visualizing STRUCTURE output and implementing the Evanno method. Conserv. Genet. Resour..

[83] Rosenberg N.A. (2004). DISTRUCT: A program for the graphical display of population structure. Mol. Ecol. Notes.

