# Supplementary material for: Comparative Transcriptome Analysis Reveals Adaptive Evolution of Notopterygium incisum and Notopterygium franchetii, Two High-Alpine Herbal Species Endemic to China
Source: Molecules. 2017 Jul 11;22(7):1158. doi: 10.3390/molecules22071158 (PMC6152189; doi:10.3390/molecules22071158)
Supplement: Supplementary file 1 [file molecules-22-01158-s001.pdf]

Supporting Information

Table S1. The information of 45 individuals used in the genetic diversity analysis.

Table S2. The annotation information of 381 positively selected genes in the NR database and the expression (RPKM) of genes in *Notopterygium incisum* and *Notopterygium franchetii*.

Table S3. The expression (RPKM) of unigenes identified by RNA-sequencing under significant enrichment analysis included GO and KEGG enrichmentsin *Notopterygium incisum* and *Notopterygium franchetii.*

Table S4. Annotated unigenes in the glutathione metabolism pathway.

Table S5. Annotated unigenes in the plant–pathogen interaction pathway.

Table S6. Characteristics of 17 SSR markers identified in this study.

Table S7. Comparison of genetic diversity between *Notopterygium incisum* and*Notopterygium franchetii* based on 17 SSR loci.

Figure S1. Top-hit species distribution of BLASTx matches to unigenes.

Figure S2. KOG function classification of unigenes.

Figure S3. A total of 3823 pairs of single-copy orthologous genes were found and used to calculate Ka/Ks ratios. Of these orthologs, 381 pairs with a Ka/Ks value > 1 were found indicating positive selection (blue dots), and 857 had a Ka/Ks ratio between 0.5 and 1, indicating weak purifying selection (green dots).

Figure S4. Gene Ontology (GO) distributions of positively selected genes.

Figure S5. Distribution of different SSR repeat motif types.

Figure S6. Bayesian inference analysis of microsatellite data for determining the most likely number of cluster (K) for the two species. The distribution of the likelihood L (K) values (A) and ΔK values (B) are presented for 1–6 (20 replicates). STRUCTURE plots are presented for best $K = 2$ and $K = 3$, respectively.

Table S1 The information of 45 individuals used in the genetic diversity analysis.

Species	Populations	Number of Individuals	Location	Coordinates	Altitude (m)
N. incisum	E	5	Guangtoushan, Shaanxi	34.05°N, 107.70°E	3190
	Q	5	Baoxing, Sichuan	30.37°N, 120.82°E	3442
	X	5	Ganzi, Sichuan	32.30°N, 100.48°E	4073
	HA	5	Tianzhu, Gansu	37.91°N, 103.25°E	3102
	HH	5	Zhouqu, Gansu	34.12°N, 104.51°E	3360
N. franchetii	KQ	5	Xinglongshan, Gansu	35.78°N, 104.03°E	2400
	KI	5	Maqu, Gansu	33.10°N, 102.07°E	2379
	YB	5	Yuzhong, Gansu	35.31°N, 104.67°E	2847
	YE	5	Tianzhu, Gansu	37.60°N, 103.40°E	2816

Table S2 The annotation information of 381 positively selected genes in the NR database and the expression (RPKM) of genes in *Notopterygium incisum* and *Notopterygium franchetii.*

gene ID	gene length		RPKM		NR-annotation
	N. franchetii	*N. incisum*	*N. franchetii*	*N. incisum*	
Unigene0000359_Nf-vs-Unigene0010073_Ni	837	679	10.126	4.296	--

Unigene0000381_Nf-vs-Unigene0010041_Ni	391	408	3.980	6.819	TIR-NB-LRR type resistance protein RGA2 [*Vitis rotundifolia*]
Unigene0000424_Nf-vs-Unigene0034533_Ni	1828	1550	38.139	12.912	hypothetical protein BVRB_5g123720 [*Beta vulgaris* subsp. *vulgaris*]
Unigene0000432_Nf-vs-Unigene0005197_Ni	1481	1380	18.012	13.722	PREDICTED: probable nucleoredoxin 1 [*Nelumbo nucifera*]
Unigene0000618_Nf-vs-Unigene0006057_Ni	1087	856	13.600	4.666	--
Unigene0001176_Nf-vs-Unigene0012093_Ni	1700	1043	6.980	5.120	PREDICTED: poly(A) RNA polymerase protein cid1-like [*Fragaria vesca* subsp. *vesca*]
Unigene0001496_Nf-vs-Unigene0028377_Ni	1479	1522	17.116	14.889	--
Unigene0001777_Nf-vs-Unigene0051692_Ni	833	533	14.077	0.926	--
Unigene0002281_Nf-vs-Unigene0015752_Ni	1273	903	31.455	10.287	PREDICTED: low-temperature-induced 65 kDa protein-like [*Populus euphratica*]
Unigene0002378_Nf-vs-Unigene0056293_Ni	1815	2125	20.806	25.932	PREDICTED: transcription factor bHLH130-like [*Vitis vinifera*]
Unigene0002439_Nf-vs-Unigene0007395_Ni	1004	1814	3.709	8.534	--
Unigene0002485_Nf-vs-Unigene0033537_Ni	3445	1192	4.420	7.755	Ubiquitin fusion degradation 1 [*Gossypium arboreum*]
Unigene0002620_Nf-vs-Unigene0019446_Ni	1005	656	4.756	7.867	PREDICTED: probable WRKY transcription factor 48 [*Vitis vinifera*]
Unigene0002695_Nf-vs-Unigene0031685_Ni	932	857	47.078	197.768	--
Unigene0002975_Nf-vs-Unigene0008092_Ni	3303	1446	29.520	25.137	PREDICTED: neurofilament heavy polypeptide-like [*Vitis vinifera*]
Unigene0003012_Nf-vs-Unigene0079253_Ni	806	360	3.896	3.116	PREDICTED: helicase protein MOM1 isoform X2 [*Vitis vinifera*]
Unigene0003166_Nf-vs-Unigene0075754_Ni	483	311	3.049	2.886	rps1 gene product (mitochondrion) [*Daucus carota* subsp. *sativus*]
Unigene0003489_Nf-vs-Unigene0003531_Ni	866	956	6.514	15.349	ethylene response factor 2.1 [*Bupleurum kaoi*]
Unigene0003573_Nf-vs-Unigene0028259_Ni	3129	1923	7.655	10.991	PREDICTED: filament-like plant protein 3 [*Sesamum indicum*]
Unigene0003842_Nf-vs-Unigene0018810_Ni	1176	2277	4.064	7.213	--
Unigene0003935_Nf-vs-Unigene0020561_Ni	1191	678	5.483	6.155	RPM1 interacting protein 4 transcript 2 [*Lactuca viminea*]
Unigene0004147_Nf-vs-Unigene0017827_Ni	719	765	2.010	0.939	PREDICTED: protein SUPPRESSOR OF npr1-1, CONSTITUTIVE 1-like [*Prunus mume*]
Unigene0004391_Nf-vs-Unigene0005865_Ni	495	578	2.077	10.093	PREDICTED: beta-galactosidase 3-like [*Gossypium raimondii*]
Unigene0004462_Nf-vs-Unigene0018800_Ni	501	467	6.267	3.171	Os11g0211800 [*Oryza sativa* Japonica Group]
Unigene0004641_Nf-vs-Unigene0003240_Ni	777	453	131.534	9.708	--
Unigene0004705_Nf-vs-Unigene0006408_Ni	1340	1224	11.053	0.000	BnaC09g48000D [*Brassica napus*]
Unigene0004720_Nf-vs-Unigene0028557_Ni	1096	1450	3.575	131.000	Polyadenylate-binding protein 1-B-binding protein [*Theobroma cacao*]
Unigene0004766_Nf-vs-Unigene0058852_Ni	318	305	5.855	7.651	PREDICTED: TMV resistance protein N-like [*Vitis vinifera*]
Unigene0004811_Nf-vs-Unigene0011950_Ni	726	982	5.320	17.182	cysteine/histidine-rich C1 domain protein [*Medicago truncatula*]
Unigene0004831_Nf-vs-Unigene0040055_Ni	1267	1125	16.273	6.661	PREDICTED: mediator of RNA polymerase II transcription subunit 36a-like [*Sesamum indicum*]
Unigene0004854_Nf-vs-Unigene0045682_Ni	855	843	24.342	12.350	PREDICTED: DNA-directed RNA polymerase V subunit 7-like [*Solanum tuberosum*]
Unigene0005102_Nf-vs-Unigene0025796_Ni	1816	1005	5.983	6.206	PREDICTED: serine/threonine-protein phosphatase 7 long form homolog [*Elaeis guineensis*]
Unigene0005121_Nf-vs-Unigene0047012_Ni	803	1140	8.790	0.000	PREDICTED: transcription factor bHLH100-like [*Fragaria vesca* subsp. *vesca*]
Unigene0005122_Nf-vs-Unigene0039904_Ni	1179	835	18.219	10.748	PREDICTED: 60 kDa jasmonate-induced protein-like [*Phoenix dactylifera*]
Unigene0005151_Nf-vs-Unigene0074297_Ni	1159	422	7.672	5.317	--
Unigene0005167_Nf-vs-Unigene0004846_Ni	576	671	17.078	197.015	BnaA10g17250D [*Brassica napus*]
Unigene0005346_Nf-vs-Unigene0004891_Ni	1030	1365	139.881	62.133	Bidirectional sugar transporter SWEET6b [*Triticum urartu*]
Unigene0005368_Nf-vs-Unigene0024473_Ni	707	676	4.952	8.099	PREDICTED: F-box only protein 11-like [*Eucalyptus grandis*]
Unigene0005369_Nf-vs-Unigene0078774_Ni	663	303	5.407	2.814	PREDICTED: zinc finger CCCH domain-containing protein 15-like [*Solanum tuberosum*]

Unigene0005590_Nf-vs-Unigene0026993_Ni	773	735	44.862	69.233	PREDICTED: nuclear transcription factor Y subunit B-3 [*Vitis vinifera*]
Unigene0005633_Nf-vs-Unigene0005004_Ni	898	1511	3.466	6.266	Chaperone protein ClpB 1 [*Morus notabilis*]
Unigene0005680_Nf-vs-Unigene0021249_Ni	446	596	3.738	3.539	peptidase M48 family protein [*Arabidopsis lyrata* subsp. *lyrata*]
Unigene0005744_Nf-vs-Unigene0002008_Ni	413	932	12.985	16.996	--
Unigene0006009_Nf-vs-Unigene0075035_Ni	314	321	17.168	8.527	PREDICTED: signaling mucin HKR1-like [*Solanum tuberosum*]
Unigene0006057_Nf-vs-Unigene0003924_Ni	965	737	11.662	6.637	Pathogenesis-related PR-1 type [*Gossypium arboreum*]
Unigene0006143_Nf-vs-Unigene0079537_Ni	899	906	11.251	75.829	--
Unigene0006146_Nf-vs-Unigene0078876_Ni	769	601	8.311	8.362	--
Unigene0006216_Nf-vs-Unigene0024658_Ni	847	719	4.823	7.240	PREDICTED: protein FAR1-RELATED SEQUENCE 9 [*Prunus mume*]
Unigene0006221_Nf-vs-Unigene0032770_Ni	997	861	4.599	0.990	F-box and associated interaction domains-containing protein [*Theobroma cacao*]
Unigene0006619_Nf-vs-Unigene0032139_Ni	2227	949	18.080	13.524	PREDICTED: uncharacterized protein LOC105786413 isoform X2 [*Gossypium raimondii*]
Unigene0006691_Nf-vs-Unigene0030381_Ni	865	851	71.701	52.414	PREDICTED: 1,4-dihydroxy-2-naphthoyl-CoA thioesterase 1 [*Gossypium raimondii*]
Unigene0006744_Nf-vs-Unigene0078346_Ni	391	320	3.767	5.469	PREDICTED: tRNA pseudouridine synthase-like 1 [*Solanum lycopersicum*]
Unigene0006880_Nf-vs-Unigene0077363_Ni	1386	424	2.687	4.128	PH01B031C15.18 [*Phyllostachys edulis*]
Unigene0006951_Nf-vs-Unigene0075020_Ni	990	301	27.563	4.174	PREDICTED: nucleolar protein 56-like [*Setaria italica*]
Unigene0007036_Nf-vs-Unigene0024943_Ni	1637	1578	5.432	8.759	Disease resistance protein (TIR-NBS class) [*Theobroma cacao*]
Unigene0007127_Nf-vs-Unigene0007900_Ni	1210	1127	37.318	9.556	PREDICTED: pheromone-processing carboxypeptidase KEX1-like [*Prunus mume*]
Unigene0007181_Nf-vs-Unigene0023096_Ni	1794	410	2.292	4.050	PREDICTED: uncharacterized protein LOC103848292 [*Brassica rapa*]
Unigene0007337_Nf-vs-Unigene0025479_Ni	645	578	4.610	4.736	PREDICTED: phosphatidylinositol N-acetylglucosaminyltransferase subunit P-like [*Solanum tuberosum*]
Unigene0007467_Nf-vs-Unigene0078437_Ni	626	564	9.810	9.150	--
Unigene0007522_Nf-vs-Unigene0030325_Ni	1009	690	3.360	7.024	Protein SMG8 [*Glycine soja*]
Unigene0007693_Nf-vs-Unigene0005813_Ni	1040	951	4.970	9.862	PREDICTED: protein At-4/1 [*Prunus mume*]
Unigene0007773_Nf-vs-Unigene0032171_Ni	1159	1326	15.128	12.995	DNAJ/Hsp40 domain-containing protein [*Solanum lycopersicum*]
Unigene0007891_Nf-vs-Unigene0031423_Ni	1633	1586	10.261	12.251	PREDICTED: F-box/kelch-repeat protein At3g06240-like isoform X1 [*Eucalyptus grandis*]
Unigene0008092_Nf-vs-Unigene0023388_Ni	723	472	77.097	4.373	PREDICTED: macrophage migration inhibitory factor homolog [*Populus euphratica*]
Unigene0008182_Nf-vs-Unigene0079025_Ni	1028	996	3.325	5.947	HECT; Ubiquitin [*Medicago truncatula*]
Unigene0008217_Nf-vs-Unigene0003538_Ni	902	956	4.005	3.239	PREDICTED: tRNA-splicing endonuclease subunit Sen54-like [*Nicotiana tomentosiformis*]
Unigene0008296_Nf-vs-Unigene0006771_Ni	824	593	4.519	4.994	PREDICTED: CTL-like protein DDB_G0288717 [*Vitis vinifera*]
Unigene0008307_Nf-vs-Unigene0001254_Ni	514	511	7.460	5.006	--
Unigene0008333_Nf-vs-Unigene0031674_Ni	1474	1106	6.787	10.062	PREDICTED: dof zinc finger protein DOF2.4-like [*Nicotiana sylvestris*]
Unigene0008436_Nf-vs-Unigene0031079_Ni	972	671	4.317	7.022	PREDICTED: protein ALWAYS EARLY 3 [*Sesamum indicum*]
Unigene0008926_Nf-vs-Unigene0025459_Ni	1150	1156	6.935	8.734	PREDICTED: cell cycle checkpoint protein RAD17 [*Vitis vinifera*]
Unigene0008932_Nf-vs-Unigene0029362_Ni	1218	1353	1.004	7.197	PREDICTED: WRKY transcription factor 22-like isoform X1 [*Vitis vinifera*]
Unigene0009122_Nf-vs-Unigene0035012_Ni	910	993	8.977	191.876	PREDICTED: probable calcium-binding protein CML45 [*Nicotiana tomentosiformis*]
Unigene0009149_Nf-vs-Unigene0022902_Ni	1016	2141	6.591	7.420	PREDICTED: zinc finger CCCH domain-containing protein 6-like [*Nelumbo nucifera*]
Unigene0009379_Nf-vs-Unigene0032928_Ni	1268	1175	0.701	2.062	BnaC01g34350D [*Brassica napus*]
Unigene0009418_Nf-vs-Unigene0050694_Ni	618	850	8.138	10.083	PREDICTED: sorbin and SH3 domain-containing protein 2-like [*Populus euphratica*]
Unigene0009424_Nf-vs-Unigene0028844_Ni	1631	824	3.765	75.152	PREDICTED: high mobility group B protein 1-like [*Nelumbo nucifera*]

Unigene0009468_Nf-vs-Unigene0034401_Ni	980	810	8.988	9.806	PREDICTED: E3 ubiquitin-protein ligase RNF4-like [*Nicotiana tomentosiformis*]
Unigene0009473_Nf-vs-Unigene0078087_Ni	571	376	7.981	4.177	OSJNBa0060B20.14 [*Oryza sativa* Japonica Group]
Unigene0009577_Nf-vs-Unigene0033342_Ni	788	906	27.047	467.061	BnaC09g40590D [*Brassica napus*]
Unigene0009612_Nf-vs-Unigene0079552_Ni	328	376	9.912	4.177	PREDICTED: uncharacterized protein LOC101247829 [*Solanum lycopersicum*]
Unigene0009739_Nf-vs-Unigene0010072_Ni	545	316	2.294	8.378	PREDICTED: FAS-associated factor 2-like [*Nicotiana sylvestris*]
Unigene0009751_Nf-vs-Unigene0004848_Ni	4226	1891	620.501	31.134	Cell wall-associated hydrolase, partial [*Medicago truncatula*]
Unigene0010079_Nf-vs-Unigene0058717_Ni	366	401	2.733	1.455	PREDICTED: DEAD-box ATP-dependent RNA helicase 56-like, partial [*Tarenaya hassleriana*]
Unigene0010130_Nf-vs-Unigene0077192_Ni	885	746	7.473	5.474	PREDICTED: WAS protein family homolog 1-like [*Eucalyptus grandis*]
Unigene0010190_Nf-vs-Unigene0005255_Ni	1191	1197	12.692	25.267	BnaCnng28270D [*Brassica napus*]
Unigene0010202_Nf-vs-Unigene0022606_Ni	1313	612	8.994	4.619	PREDICTED: 60 kDa jasmonate-induced protein-like [*Phoenix dactylifera*]
Unigene0010716_Nf-vs-Unigene0077789_Ni	1688	559	13.186	6.181	PREDICTED: protein IQ-DOMAIN 14 [*Jatropha curcas*]
Unigene0010774_Nf-vs-Unigene0033350_Ni	993	809	35.231	11.870	BnaC08g11630D [*Brassica napus*]
Unigene0011066_Nf-vs-Unigene0028863_Ni	894	595	3.108	1.810	23.6 kDa heat shock, mitochondrial -like protein [*Gossypium arboreum*]
Unigene0011195_Nf-vs-Unigene0025035_Ni	1418	530	10.308	3.471	transferase family protein [*Populus trichocarpa*]
Unigene0011222_Nf-vs-Unigene0028005_Ni	816	733	260.370	122.928	Mycosubtilin synthase subunit A [*Gossypium arboreum*]
Unigene0011240_Nf-vs-Unigene0016074_Ni	848	1213	1.016	10.247	--
Unigene0011257_Nf-vs-Unigene0025659_Ni	595	867	3.736	4.141	smek1 [*Gossypium arboreum*]
Unigene0011275_Nf-vs-Unigene0032709_Ni	887	867	35.557	3.468	PREDICTED: ORM1-like protein 2 [*Sesamum indicum*]
Unigene0011398_Nf-vs-Unigene0038185_Ni	689	719	44.645	30.956	PREDICTED: mitochondrial import inner membrane translocase subunit TIM22-2-like [*Solanum tuberosum*]
Unigene0011491_Nf-vs-Unigene0025042_Ni	790	917	3.236	9.053	PREDICTED: mediator of RNA polymerase II transcription subunit 19a-like isoform X2 [*Musa acuminata* subsp. *malaccensis*]
Unigene0011522_Nf-vs-Unigene0023230_Ni	1482	626	4.950	5.376	PREDICTED: F-box/LRR-repeat protein At3g59190 isoform X1 [*Nicotiana tomentosiformis*]
Unigene0011590_Nf-vs-Unigene0034366_Ni	1396	1506	18.950	103.185	PREDICTED: RING-H2 finger protein ATL3 [*Sesamum indicum*]
Unigene0011606_Nf-vs-Unigene0037746_Ni	660	725	24.335	31.752	PREDICTED: protein yippee-like [*Prunus mume*]
Unigene0011894_Nf-vs-Unigene0022472_Ni	1092	1051	577.198	142.733	PREDICTED: dirigent protein 22-like [*Nicotiana tomentosiformis*]
Unigene0011901_Nf-vs-Unigene0003584_Ni	576	341	23.060	4.474	--
Unigene0012430_Nf-vs-Unigene0046109_Ni	717	1163	60.613	60.191	PREDICTED: aspartate aminotransferase, mitochondrial [*Nicotiana sylvestris*]
Unigene0012558_Nf-vs-Unigene0027538_Ni	726	552	27.558	11.706	PREDICTED: chitin-binding lectin 1 [*Vitis vinifera*]
Unigene0012569_Nf-vs-Unigene0041572_Ni	1050	1154	8.839	13.960	PREDICTED: GATA transcription factor 1 [*Sesamum indicum*]
Unigene0012570_Nf-vs-Unigene0041570_Ni	1072	938	0.726	0.383	PREDICTED: GATA transcription factor 1 [*Sesamum indicum*]
Unigene0012630_Nf-vs-Unigene0048307_Ni	1207	1115	0.000	30.466	PREDICTED: haloacid dehalogenase-like hydrolase domain-containing protein 3 [*Sesamum indicum*]
Unigene0012751_Nf-vs-Unigene0007658_Ni	912	1093	6.642	0.616	--
Unigene0012908_Nf-vs-Unigene0030132_Ni	1811	1817	26.621	7.063	PREDICTED: tubulin-folding cofactor E [*Sesamum indicum*]
Unigene0013031_Nf-vs-Unigene0022740_Ni	981	639	62.769	92.907	spindle pole body component protein [*Gossypium arboreum*]
Unigene0013147_Nf-vs-Unigene0032574_Ni	616	938	7.624	219.871	phloem protein 2-2 [*Apium graveolens* Dulce Group]
Unigene0013197_Nf-vs-Unigene0039423_Ni	646	660	45.509	4.011	CM0545.370.nc [*Lotus japonicus*]
Unigene0013279_Nf-vs-Unigene0024810_Ni	911	922	52.830	26.184	Immunoglobulin superfamily member 2 [*Gossypium arboreum*]
Unigene0013291_Nf-vs-Unigene0030591_Ni	964	666	6.053	6.266	--
Unigene0013304_Nf-vs-Unigene0029842_Ni	1146	1181	6.692	13.641	PREDICTED: vesicle-associated protein 1-1-like [Sesamum indicum]
Unigene0013616_Nf-vs-Unigene0006765_Ni	1802	4991	6.399	7.516	PREDICTED: protein SCAR2 [*Vitis vinifera*]

Unigene0013627_Nf-vs-Unigene0042684_Ni	1097	1084	15.477	8.279	PREDICTED: transcription factor bHLH81-like isoform X1 [Solanum tuberosum]
Unigene0013873_Nf-vs-Unigene0000246_Ni	945	812	7.557	5.361	PREDICTED: helicase protein MOM1-like [*Jatropha curcas*]
Unigene0014184_Nf-vs-Unigene0027922_Ni	1193	1269	19.099	85.999	PREDICTED: 29 kDa ribonucleoprotein A, chloroplastic [*Jatropha curcas*]
Unigene0014404_Nf-vs-Unigene0064657_Ni	1080	1076	29.949	0.083	RNA-binding protein with multiple splicing [*Morella rubra*]
Unigene0014419_Nf-vs-Unigene0041838_Ni	1239	1219	21.530	20.946	ribosomal protein L2 (mitochondrion) [*Vaccinium macrocarpon*]
Unigene0014452_Nf-vs-Unigene0006924_Ni	709	745	79.129	117.936	von Willebrand factor A domain-containing 2 [*Gossypium arboreum*]
Unigene0014820_Nf-vs-Unigene0080807_Ni	985	411	5.614	4.476	PREDICTED: F-box protein At2g26160-like [*Jatropha curcas*]
Unigene0014905_Nf-vs-Unigene0024985_Ni	797	993	20.745	66.836	PREDICTED: uncharacterized protein LOC102603344 isoform X1 [*Solanum tuberosum*]
Unigene0014932_Nf-vs-Unigene0026084_Ni	662	569	36.938	22.476	PREDICTED: MFP1 attachment factor 1-like [*Vitis vinifera*]
Unigene0014984_Nf-vs-Unigene0004575_Ni	1384	1330	13.874	12.686	--
Unigene0015027_Nf-vs-Unigene0077505_Ni	532	410	67.797	14.119	Os01g0811200 [*Oryza sativa* Japonica Group]
Unigene0015059_Nf-vs-Unigene0026355_Ni	754	781	18.685	15.858	PREDICTED: SOSS complex subunit B homolog [*Eucalyptus grandis*]
Unigene0015112_Nf-vs-Unigene0016456_Ni	1935	1372	8.760	2.911	Translation initiation factor IF-3 [*Glycine soja*]
Unigene0015120_Nf-vs-Unigene0066691_Ni	1160	704	12.672	1.530	dihydroflavonol-4-reductase [*Nicotiana tabacum*]
Unigene0015511_Nf-vs-Unigene0019013_Ni	2571	371	14.418	3.024	hydrolase family protein [*Populus trichocarpa*]
Unigene0015629_Nf-vs-Unigene0019178_Ni	1006	344	2.928	3.653	PREDICTED: pentatricopeptide repeat-containing protein At1g26900, mitochondrial [*Fragaria vesca* subsp. *vesca*]
Unigene0015642_Nf-vs-Unigene0005521_Ni	821	315	0.575	4.986	--
Unigene0015674_Nf-vs-Unigene0022918_Ni	831	875	20.297	69.439	PREDICTED: CASP-like protein 1 [*Sesamum indicum*]
Unigene0015880_Nf-vs-Unigene0079501_Ni	852	612	19.797	4.693	--
Unigene0015923_Nf-vs-Unigene0030699_Ni	1914	969	7.041	7.456	Ribulose-1,5 bisphosphate carboxylase/oxygenase large subunit N-methyltransferase, chloroplastic [*Gossypium arboreum*]
Unigene0016130_Nf-vs-Unigene0000151_Ni	1461	1423	18.316	10.911	PREDICTED: mediator-associated protein 1-like [*Nelumbo nucifera*]
Unigene0016152_Nf-vs-Unigene0041581_Ni	1524	1518	9.718	21.136	PREDICTED: L-type lectin-domain containing receptor kinase VIII.2-like [*Nicotiana sylvestris*]
Unigene0016215_Nf-vs-Unigene0020934_Ni	965	1034	7.976	29.988	PREDICTED: pathogen-related protein-like [*Populus euphratica*]
Unigene0016240_Nf-vs-Unigene0039314_Ni	959	1014	12.836	120.061	PREDICTED: suppressor protein SRP40-like [*Pyrus* x *bretschneideri*]
Unigene0016408_Nf-vs-Unigene0032214_Ni	967	970	20.029	131.012	BnaC04g24950D [*Brassica napus*]
Unigene0016538_Nf-vs-Unigene0037904_Ni	990	823	9.038	14.340	Late embryogenesis abundant hydroxyproline-rich glycofamily protein [*Theobroma cacao*]
Unigene0016621_Nf-vs-Unigene0029733_Ni	1243	1253	28.838	100.849	thioredoxin-like 7 family protein [*Populus trichocarpa*]
Unigene0016873_Nf-vs-Unigene0015294_Ni	1480	1418	1.183	2.152	Serine/arginine-rich splicing factor 12 [*Gossypium arboreum*]
Unigene0016874_Nf-vs-Unigene0007638_Ni	1152	1090	2.460	0.247	PREDICTED: serine/arginine-rich SC35-like splicing factor SCL30 [*Gossypium raimondii*]
Unigene0016981_Nf-vs-Unigene0005097_Ni	694	566	52.492	11.258	--
Unigene0017282_Nf-vs-Unigene0021275_Ni	3019	1454	6.351	6.574	PREDICTED: probable ubiquitin-like-specific protease 2B-like [*Citrus sinensis*]
Unigene0017347_Nf-vs-Unigene0074278_Ni	533	394	11.157	3.303	PREDICTED: WAS/WASL-interacting protein family member 3-like [*Nicotiana tomentosiformis*]
Unigene0017433_Nf-vs-Unigene0031789_Ni	1098	942	6.251	10.051	PREDICTED: B-cell receptor-associated protein 31-like [*Glycine max*]
Unigene0017964_Nf-vs-Unigene0025492_Ni	739	688	8.348	24.524	phloem protein 2-2 [*Apium graveolens* Dulce Group]
Unigene0017982_Nf-vs-Unigene0036028_Ni	1173	1154	11.039	7.155	PREDICTED: biotin carboxyl carrier protein of acetyl-CoA carboxylase 2, chloroplastic isoform X1 [*Vitis vinifera*]
Unigene0018133_Nf-vs-Unigene0043913_Ni	648	928	4.417	39.458	PREDICTED: rRNA biogenesis protein rrp36-like [*Malus domestica*]

Unigene0018180_Nf-vs-Unigene0030308_Ni	717	443	8.216	2.431	RNA-binding S4 domain-containing protein [*Theobroma cacao*]
Unigene0018373_Nf-vs-Unigene0016559_Ni	1074	2656	8.978	10.796	PREDICTED: zinc finger CCCH domain-containing protein 38-like isoform X3 [*Gossypium raimondii*]
Unigene0018587_Nf-vs-Unigene0027691_Ni	5152	598	5.334	4.127	Tat-binding-7-like protein [*Morus notabilis*]
Unigene0018628_Nf-vs-Unigene0007188_Ni	1236	1151	7.959	3.977	PREDICTED: cysteine string protein-like isoform X1 [*Solanum tuberosum*]
Unigene0018637_Nf-vs-Unigene0038118_Ni	921	1656	10.892	20.134	PREDICTED: CBL-interacting serine/threonine-protein kinase 5-like [*Sesamum indicum*]
Unigene0018671_Nf-vs-Unigene0006969_Ni	2185	1106	13.302	8.520	PREDICTED: zinc finger protein JACKDAW-like [*Nicotiana sylvestris*]
Unigene0018771_Nf-vs-Unigene0035948_Ni	1441	1318	6.884	3.575	PREDICTED: LRR repeats and ubiquitin-like domain-containing protein At2g30105 [*Nicotiana sylvestris*]
Unigene0018945_Nf-vs-Unigene0038802_Ni	841	879	20.684	186.998	RecName: Full=Late embryogenesis abundant protein Dc3 [*Daucus carota*]
Unigene0019029_Nf-vs-Unigene0024576_Ni	1831	1153	13.143	6.889	PREDICTED: ras GTPase-activating protein-binding protein 2-like [*Sesamum indicum*]
Unigene0019413_Nf-vs-Unigene0048179_Ni	1345	1210	7.355	4.710	PREDICTED: serine/threonine-protein kinase 19 isoform X1 [*Vitis vinifera*]
Unigene0019731_Nf-vs-Unigene0049364_Ni	2003	1951	6.201	28.405	Histone [*Morus notabilis*]
Unigene0019733_Nf-vs-Unigene0005206_Ni	2003	573	6.201	11.669	Ralf-like 32 [*Theobroma cacao*]
Unigene0019980_Nf-vs-Unigene0056587_Ni	488	763	6.434	0.823	BnaA07g38220D [*Brassica napus*]
Unigene0019994_Nf-vs-Unigene0080190_Ni	661	356	100.766	2.647	PREDICTED: serine/arginine repetitive matrix protein 2-like [*Nicotiana tomentosiformis*]
Unigene0020025_Nf-vs-Unigene0025945_Ni	1051	1080	33.472	38.766	PREDICTED: cysteine and histidine-rich domain-containing protein RAR1 [*Nicotiana tomentosiformis*]
Unigene0020092_Nf-vs-Unigene0060713_Ni	701	649	4.519	1.798	PREDICTED: acetyl-coenzyme A synthetase, chloroplastic/glyoxysomal-like isoform X2 [*Nelumbo nucifera*]
Unigene0020113_Nf-vs-Unigene0013056_Ni	459	472	4.419	13.405	PREDICTED: probable disease resistance RPP8-like protein 2 [*Vitis vinifera*]
Unigene0020175_Nf-vs-Unigene0000939_Ni	835	722	30.250	6.402	PREDICTED: uncharacterized serine-rich protein C215.13-like [*Nelumbo nucifera*]
Unigene0020262_Nf-vs-Unigene0011259_Ni	1330	1773	0.919	0.405	PREDICTED: rRNA biogenesis protein RRP36 [*Populus euphratica*]
Unigene0020265_Nf-vs-Unigene0035192_Ni	1279	1287	0.044	0.000	PREDICTED: myb-like protein X [*Nicotiana tomentosiformis*]
Unigene0020280_Nf-vs-Unigene0079678_Ni	881	721	16.748	6.722	PREDICTED: leucine-rich repeat extensin-like protein 1 [*Brassica rapa*]
Unigene0020426_Nf-vs-Unigene0013897_Ni	789	613	3.029	10.175	Plastid division protein PDV1 [*Theobroma cacao*]
Unigene0020451_Nf-vs-Unigene0012937_Ni	897	884	3.779	1.777	PREDICTED: DDB1- and CUL4-associated factor 4 isoform X1 [*Nicotiana tomentosiformis*]
Unigene0020455_Nf-vs-Unigene0062953_Ni	2553	2085	0.261	1.313	RNA-binding family protein isoform 1 [*Theobroma cacao*]
Unigene0020480_Nf-vs-Unigene0038517_Ni	977	1050	50.199	7.735	--
Unigene0020512_Nf-vs-Unigene0017891_Ni	1146	1139	1.164	6.579	PREDICTED: transcription factor MYB3-like [*Sesamum indicum*]
Unigene0020774_Nf-vs-Unigene0044046_Ni	1033	1165	32.495	132.579	Os08g0107100 [*Oryza sativa* Japonica Group]
Unigene0021083_Nf-vs-Unigene0048923_Ni	1791	1743	57.157	45.234	PREDICTED: mediator-associated protein 1 [*Cucumis melo*]
Unigene0021227_Nf-vs-Unigene0041621_Ni	922	917	18.475	87.251	PREDICTED: auxin-induced protein 10A5 [*Solanum lycopersicum*]
Unigene0021400_Nf-vs-Unigene0055074_Ni	769	792	48.348	272.414	PREDICTED: protein CURVATURE THYLAKOID 1A, chloroplastic-like [*Solanum lycopersicum*]
Unigene0021987_Nf-vs-Unigene0034982_Ni	1297	1306	35.329	47.107	protein ZCF37 [*Arabidopsis thaliana*]
Unigene0022197_Nf-vs-Unigene0074218_Ni	814	568	8.295	6.557	PREDICTED: mitochondrial import inner membrane translocase subunit TIM22 [*Gossypium raimondii*]
Unigene0022206_Nf-vs-Unigene0031466_Ni	1189	1200	206.384	77.369	plant cadmium resistance 8 -like protein [*Gossypium arboreum*]
Unigene0022416_Nf-vs-Unigene0000008_Ni	945	936	10.939	14.766	BnaC01g10500D [*Brassica napus*]

Unigene0022621_Nf-vs-Unigene0072622_Ni	824	845	8.498	6.691	PREDICTED: S phase cyclin A-associated protein in the endoplasmic reticulum-like [*Sesamum indicum*]
Unigene0022734_Nf-vs-Unigene0013597_Ni	681	791	9.467	7.261	fluorescent in blue light, chloroplastic -like protein [*Gossypium arboreum*]
Unigene0022738_Nf-vs-Unigene0004434_Ni	1629	1566	36.999	10.774	hypothetical protein BVRB_5g123720 [*Beta vulgaris* subsp. *vulgaris*]
Unigene0022818_Nf-vs-Unigene0035753_Ni	1467	1677	0.265	6.636	PREDICTED: microtubule-associated protein futsch-like isoform X2 [*Nicotiana tomentosiformis*]
Unigene0022927_Nf-vs-Unigene0060281_Ni	1384	1410	13.352	22.914	PREDICTED: methyl-CpG-binding domain-containing protein 11-like [*Malus domestica*]
Unigene0022946_Nf-vs-Unigene0013074_Ni	1456	890	13.016	4.387	kelch repeat-containing F-box family protein [*Arabidopsis lyrata* subsp. *lyrata*]
Unigene0023567_Nf-vs-Unigene0061487_Ni	517	421	0.806	17.054	PREDICTED: remorin-like [*Camelina sativa*]
Unigene0023727_Nf-vs-Unigene0046539_Ni	2159	2111	19.087	12.584	PHD-finger family protein expressed [*Medicago truncatula*]
Unigene0023741_Nf-vs-Unigene0073519_Ni	1600	1659	6.356	3.787	PREDICTED: G-type lectin S-receptor-like serine/threonine-protein kinase At2g19130 [*Cicer arietinum*]
Unigene0023853_Nf-vs-Unigene0064218_Ni	3376	3238	15.491	12.847	hypothetical salt-inducible protein [*Prunus mume*]
Unigene0023937_Nf-vs-Unigene0007252_Ni	1050	1163	17.122	65.863	PREDICTED: eukaryotic translation initiation factor 4E-1-like isoform X1 [*Glycine max*]
Unigene0024420_Nf-vs-Unigene0029761_Ni	861	854	6.616	18.706	PREDICTED: NAC transcription factor ONAC010-like isoform X1 [*Citrus sinensis*]
Unigene0024513_Nf-vs-Unigene0017606_Ni	317	523	7.714	6.692	hypothetical protein Csa_3G020010 [*Cucumis sativus*]
Unigene0024548_Nf-vs-Unigene0000760_Ni	2717	2715	12.641	12.363	PREDICTED: probable C-terminal domain small phosphatase isoform X2 [*Populus euphratica*]
Unigene0024569_Nf-vs-Unigene0003348_Ni	1457	1547	15.734	15.403	--
Unigene0024574_Nf-vs-Unigene0048032_Ni	983	853	0.057	1.368	PREDICTED: luc7-like protein [*Musa acuminata* subsp. *malaccensis*]
Unigene0024793_Nf-vs-Unigene0010919_Ni	862	921	1258.264	989.019	PREDICTED: S-norcoclaurine synthase-like [*Sesamum indicum*]
Unigene0025580_Nf-vs-Unigene0033531_Ni	1342	1260	25.096	151.964	PREDICTED: 60 kDa jasmonate-induced protein-like [*Elaeis guineensis*]
Unigene0025656_Nf-vs-Unigene0019428_Ni	685	921	6.044	242.054	--
Unigene0025679_Nf-vs-Unigene0036558_Ni	744	833	17435.103	84.629	PREDICTED: pathogenesis-related protein PR-1 type-like [*Phoenix dactylifera*]
Unigene0025806_Nf-vs-Unigene0015375_Ni	2889	912	84.949	10.431	importin alpha [*Galdieria sulphuraria*]
Unigene0026399_Nf-vs-Unigene0044561_Ni	1809	1767	6.144	22.602	BnaC04g13350D [*Brassica napus*]
Unigene0026812_Nf-vs-Unigene0036682_Ni	1105	1074	9.028	40.445	PREDICTED: REF/SRPP-like protein At3g05500 [*Populus euphratica*]
Unigene0026960_Nf-vs-Unigene0010644_Ni	1059	1015	7.426	2.653	PREDICTED: 60 kDa jasmonate-induced protein-like [*Phoenix dactylifera*]
Unigene0027060_Nf-vs-Unigene0022456_Ni	635	798	56517.097	13062.175	plant defensin [*Bupleurum kaoi*]
Unigene0027094_Nf-vs-Unigene0003955_Ni	775	734	4.769	22.315	Transcription factor bHLH61 isoform 1 [*Theobroma cacao*]
Unigene0027123_Nf-vs-Unigene0033558_Ni	347	956	0.320	8.355	PREDICTED: LOB domain-containing protein 21 [*Vitis vinifera*]
Unigene0027312_Nf-vs-Unigene0037072_Ni	1085	2028	6.915	8.873	--
Unigene0027342_Nf-vs-Unigene0032017_Ni	626	644	18.466	0.418	--
Unigene0027376_Nf-vs-Unigene0033588_Ni	762	1021	18.343	16.481	PREDICTED: U1 small nuclear ribonucleoprotein A [*Fragaria vesca* subsp. *vesca*]
Unigene0027705_Nf-vs-Unigene0050567_Ni	1298	1411	1.606	8.682	glutathione peroxidase 2 [*Panax ginseng*]
Unigene0027867_Nf-vs-Unigene0014687_Ni	5121	4127	6.962	13.363	Ty3/gypsy retrotransposon protein [*Beta vulgaris* subsp. *vulgaris*]
Unigene0028208_Nf-vs-Unigene0019579_Ni	776	356	1.611	5.798	Nucleoporin NUP188-like protein [*Medicago truncatula*]
Unigene0028247_Nf-vs-Unigene0034922_Ni	376	624	6.282	9.708	nematode resistance-like protein [*Prunus cerasifera*]
Unigene0028678_Nf-vs-Unigene0029087_Ni	904	767	2.705	17.844	NC domain-containing protein-related [*Theobroma cacao*]
Unigene0028686_Nf-vs-Unigene0074522_Ni	659	344	3.331	3.783	PREDICTED: protein SUPPRESSOR OF GENE SILENCING 3-like [*Prunus mume*]
Unigene0028862_Nf-vs-Unigene0027192_Ni	702	522	93.931	54.931	Inhibitor of trypsin and hageman factor [*Medicago truncatula*]

Unigene0028994_Nf-vs-Unigene0010944_Ni	1458	1361	0.953	31.191	PREDICTED: NHL repeat-containing protein 2 isoform X2 [*Beta vulgaris* subsp. *vulgaris*]
Unigene0029347_Nf-vs-Unigene0064689_Ni	1262	1109	12.727	0.162	PREDICTED: transcription factor MYB48 isoform X1 [*Eucalyptus grandis*]
Unigene0029368_Nf-vs-Unigene0021122_Ni	757	741	0.073	325.863	PREDICTED: late embryogenesis abundant protein Lea5 [*Nicotiana tomentosiformis*]
Unigene0029448_Nf-vs-Unigene0053413_Ni	1030	1039	13.786	17.621	PREDICTED: glutathione S-transferase T1 [*Cucumis sativus*]
Unigene0029481_Nf-vs-Unigene0081439_Ni	787	506	4.978	5.498	PREDICTED: aspartic proteinase CDR1-like [*Eucalyptus grandis*]
Unigene0029652_Nf-vs-Unigene0001997_Ni	1121	1194	4.858	9.809	PREDICTED: F-box protein At3g56470-like [*Vitis vinifera*]
Unigene0029802_Nf-vs-Unigene0016160_Ni	993	701	1.091	16.964	PREDICTED: nucleolar protein 56-like [*Setaria italica*]
Unigene0030019_Nf-vs-Unigene0014662_Ni	1059	805	2.467	1.282	PREDICTED: RNA-binding protein 39-like [*Camelina sativa*]
Unigene0030148_Nf-vs-Unigene0027122_Ni	1535	1533	9.902	13.524	--
Unigene0030230_Nf-vs-Unigene0009205_Ni	1350	1221	9.262	10.584	PREDICTED: probable nucleoredoxin 1 [*Cucumis sativus*]
Unigene0030346_Nf-vs-Unigene0023404_Ni	1208	423	3.174	2.652	PREDICTED: F-box protein SKIP23-like [*Nicotiana tomentosiformis*]
Unigene0030403_Nf-vs-Unigene0045355_Ni	1208	1093	1.426	1.232	PREDICTED: 60 kDa jasmonate-induced protein-like [*Beta vulgaris* subsp. *vulgaris*]
Unigene0030415_Nf-vs-Unigene0035015_Ni	1226	1249	10.585	68.047	PREDICTED: tRNA 2'-phosphotransferase 1-like isoform X1 [*Glycine max*]
Unigene0030416_Nf-vs-Unigene0040037_Ni	625	616	2379.619	227.500	PREDICTED: metallothionein-like protein 1 [*Sesamum indicum*]
Unigene0030664_Nf-vs-Unigene0068695_Ni	1295	1970	12.917	23.986	PREDICTED: probable nucleoredoxin 1 [*Beta vulgaris* subsp. *vulgaris*]
Unigene0030886_Nf-vs-Unigene0069980_Ni	613	688	18.903	6.653	PREDICTED: probable cyclic nucleotide-gated ion channel 17-like, partial [*Solanum tuberosum*]
Unigene0032125_Nf-vs-Unigene0077287_Ni	1572	744	13.346	5.066	PREDICTED: probable GPI-anchored adhesin-like protein PGA55 [*Prunus mume*]
Unigene0032340_Nf-vs-Unigene0069548_Ni	474	498	4.104	8.470	PREDICTED: kinesin-like protein KIF22 [*Malus domestica*]
Unigene0032395_Nf-vs-Unigene0047394_Ni	2040	1083	2.057	10.980	PREDICTED: leucine-rich repeat-containing protein 1-like [*Eucalyptus grandis*]
Unigene0032756_Nf-vs-Unigene0029206_Ni	1009	1019	9.942	174.210	Dcarg-1 [*Daucus carota*]
Unigene0033388_Nf-vs-Unigene0034751_Ni	559	609	6.711	2.284	--
Unigene0033641_Nf-vs-Unigene0012478_Ni	1002	942	2.579	3.859	PREDICTED: protein SUPPRESSOR OF npr1-1, CONSTITUTIVE 1-like [*Prunus mume*]
Unigene0033689_Nf-vs-Unigene0010067_Ni	1077	827	8.024	0.977	TMV resistance protein N [*Medicago truncatula*]
Unigene0033894_Nf-vs-Unigene0029517_Ni	1585	1541	9.485	8.357	PREDICTED: double-stranded RNA-binding protein 1-like [*Jatropha curcas*]
Unigene0033972_Nf-vs-Unigene0078562_Ni	705	622	10.484	7.070	PREDICTED: josephin-like protein [*Gossypium raimondii*]
Unigene0033981_Nf-vs-Unigene0011936_Ni	692	1061	4.979	4.229	PREDICTED: serine-threonine kinase receptor-associated protein-like [*Cucumis melo*]
Unigene0034520_Nf-vs-Unigene0031331_Ni	1743	601	4.320	2.240	PREDICTED: U11/U12 small nuclear ribonucleoprotein 48 kDa protein [*Nicotiana sylvestris*]
Unigene0034629_Nf-vs-Unigene0028652_Ni	399	1787	2.229	16.850	--
Unigene0034858_Nf-vs-Unigene0026742_Ni	529	774	8.352	178.450	ATFP4-like protein [*Medicago truncatula*]
Unigene0035206_Nf-vs-Unigene0006288_Ni	823	309	3.714	4.792	PREDICTED: WEB family protein At2g38370 [*Sesamum indicum*]
Unigene0035222_Nf-vs-Unigene0036561_Ni	1198	1238	2.551	7.104	PREDICTED: cell division cycle-associated protein 7-like [*Sesamum indicum*]
Unigene0035981_Nf-vs-Unigene0074523_Ni	396	582	2.035	72.399	Glutaredoxin [*Medicago truncatula*]
Unigene0036389_Nf-vs-Unigene0017317_Ni	1017	1114	9.181	9.909	--
Unigene0036652_Nf-vs-Unigene0007788_Ni	589	375	3.161	3.231	PREDICTED: vacuolar protein sorting-associated protein 8 homolog isoform X2 [*Prunus mume*]
Unigene0036899_Nf-vs-Unigene0002763_Ni	879	865	20.042	108.163	PREDICTED: ethylene-responsive transcription factor 13-like [*Solanum lycopersicum*]

Unigene0036967_Nf-vs-Unigene0028332_Ni	501	842	3.716	13.590	PREDICTED: mediator of RNA polymerase II transcription subunit 30-like isoform X2 [*Nelumbo nucifera*]
Unigene0037060_Nf-vs-Unigene0027693_Ni	477	2068	3.728	142.910	PREDICTED: SKP1-like protein 1A [*Nicotiana tomentosiformis*]
Unigene0037404_Nf-vs-Unigene0026971_Ni	1155	1103	32.046	47.396	HEAT repeat-containing 8 [*Gossypium arboreum*]
Unigene0037488_Nf-vs-Unigene0044851_Ni	1715	3487	15.943	1.699	PREDICTED: pentatricopeptide repeat-containing protein At4g35850, mitochondrial [*Sesamum indicum*]
Unigene0037697_Nf-vs-Unigene0030634_Ni	321	591	1.558	6.226	--
Unigene0038247_Nf-vs-Unigene0016547_Ni	303	723	1.834	18.061	--
Unigene0038427_Nf-vs-Unigene0018209_Ni	703	489	2.293	3.762	--
Unigene0038549_Nf-vs-Unigene0006439_Ni	308	300	2.256	4.936	--
Unigene0038563_Nf-vs-Unigene0014327_Ni	386	454	2.808	3.558	PREDICTED: phosphoinositide 3-kinase regulatory subunit 4 [*Prunus mume*]
Unigene0038631_Nf-vs-Unigene0021256_Ni	994	642	5.535	5.242	PREDICTED: LOW QUALITY PROTEIN: probable nucleoredoxin 1 [*Phoenix dactylifera*]
Unigene0038678_Nf-vs-Unigene0027510_Ni	643	607	4.019	13.307	C5a peptidase [*Gossypium arboreum*]
Unigene0039022_Nf-vs-Unigene0077214_Ni	535	1012	4.778	169.562	Desiccation-related PCC3-06 [*Gossypium arboreum*]
Unigene0039186_Nf-vs-Unigene0045935_Ni	411	693	3.516	3.820	WD repeat and FYVE domain-containing protein 3 [*Glycine soja*]
Unigene0039391_Nf-vs-Unigene0024739_Ni	420	559	2.514	63.578	Calcium-transporting ATPase 9, plasma membrane-type -like protein [*Gossypium arboreum*]
Unigene0039683_Nf-vs-Unigene0027706_Ni	747	766	3.422	7.850	PREDICTED: F-box protein CPR30-like isoform X2 [*Populus euphratica*]
Unigene0039810_Nf-vs-Unigene0001851_Ni	772	704	50.032	11.601	--
Unigene0039940_Nf-vs-Unigene0014488_Ni	337	496	2.061	6.152	--
Unigene0040154_Nf-vs-Unigene0030947_Ni	1440	1397	3.898	0.161	PREDICTED: ankyrin repeat-containing protein At5g02620 [*Vitis vinifera*]
Unigene0040165_Nf-vs-Unigene0079458_Ni	390	402	1.924	3.907	PREDICTED: pentatricopeptide repeat-containing protein At4g21705, mitochondrial-like [*Solanum lycopersicum*]
Unigene0040453_Nf-vs-Unigene0024617_Ni	723	656	8.878	9.645	Ephrin-A3 [*Gossypium arboreum*]
Unigene0040694_Nf-vs-Unigene0062806_Ni	300	629	1.853	4.495	PREDICTED: GRIP and coiled-coil domain-containing protein 2 isoform X5 [*Vitis vinifera*]
Unigene0040733_Nf-vs-Unigene0021739_Ni	920	558	4.198	6.192	Monofunctional biosynthetic peptidoglycan transglycosylase [*Gossypium arboreum*]
Unigene0041351_Nf-vs-Unigene0035051_Ni	772	916	51.363	72.062	PREDICTED: peptide methionine sulfoxide reductase B5-like [*Sesamum indicum*]
Unigene0041370_Nf-vs-Unigene0076413_Ni	565	388	4.771	5.436	PREDICTED: suppressor protein SRP40-like [*Citrus sinensis*]
Unigene0041407_Nf-vs-Unigene0045089_Ni	1504	1972	6.818	15.110	PREDICTED: probable nucleoredoxin 1-1 [*Elaeis guineensis*]
Unigene0041941_Nf-vs-Unigene0035620_Ni	1338	1355	6.874	9.306	L-type lectin-domain containing receptor kinase S.6 [*Glycine soja*]
Unigene0042075_Nf-vs-Unigene0009448_Ni	575	935	2.996	7.967	PREDICTED: glycerophosphodiester phosphodiesterase protein kinase domain-containing GDPDL2-like [*Vitis vinifera*]
Unigene0042362_Nf-vs-Unigene0034088_Ni	1307	1246	47.496	28.055	PREDICTED: transmembrane protein 64-like [*Cicer arietinum*]
Unigene0042386_Nf-vs-Unigene0039407_Ni	904	982	3.197	3.199	PREDICTED: BTB/POZ and MATH domain-containing protein 1-like isoform X2 [*Musa acuminata* subsp. *malaccensis*]
Unigene0042415_Nf-vs-Unigene0029396_Ni	715	733	10.415	9.428	PREDICTED: cold-regulated 413 plasma membrane protein 4-like [*Eucalyptus grandis*]
Unigene0042716_Nf-vs-Unigene0004531_Ni	571	884	4.574	9.391	PREDICTED: HMG-Y-related protein A-like [*Gossypium raimondii*]
Unigene0042895_Nf-vs-Unigene0025898_Ni	743	775	3.142	5.906	Triglyceride lipases,triglyceride lipases isoform 3 [*Theobroma cacao*]
Unigene0043049_Nf-vs-Unigene0007871_Ni	507	599	3.288	8.390	GCN5-related N-acetyltransferase family protein [*Populus trichocarpa*]
Unigene0043066_Nf-vs-Unigene0080802_Ni	806	933	5.413	13.467	--
Unigene0043180_Nf-vs-Unigene0022852_Ni	686	567	3.119	6.015	PREDICTED: probable apyrase 7 [*Sesamum indicum*]

Unigene0043212_Nf-vs-Unigene0027713_Ni	730	813	21.621	5646.883	RecName: Full=Non-specific lipid-transfer protein; AltName: Full=Allergen Api g 2.0101; AltName: Allergen=Api g 2; Flags: Precursor [*Apium graveolens*]
Unigene0043298_Nf-vs-Unigene0013098_Ni	491	2247	2.547	8.787	PREDICTED: CCA tRNA nucleotidyltransferase 1, mitochondrial-like [*Malus domestica*]
Unigene0043394_Nf-vs-Unigene0019417_Ni	317	416	2.980	7.012	--
Unigene0043422_Nf-vs-Unigene0012103_Ni	634	1518	2.980	4.168	Os01g0755100 [*Oryza sativa* Japonica Group]
Unigene0043572_Nf-vs-Unigene0025561_Ni	763	437	2.914	0.000	PREDICTED: sphingomyelin phosphodiesterase 4 [*Vitis vinifera*]
Unigene0043758_Nf-vs-Unigene0020511_Ni	693	440	2.727	5.405	PREDICTED: DNA mismatch repair protein MSH4, partial [*Vitis vinifera*]
Unigene0044058_Nf-vs-Unigene0022449_Ni	329	303	2.534	3.554	--
Unigene0044074_Nf-vs-Unigene0078600_Ni	456	332	1.950	2.838	PREDICTED: kinesin-like protein 3 [*Beta vulgaris* subsp. *vulgaris*]
Unigene0044119_Nf-vs-Unigene0013204_Ni	1003	983	3.962	7.578	--
Unigene0044309_Nf-vs-Unigene0028145_Ni	648	822	14.065	261.216	--
Unigene0044352_Nf-vs-Unigene0002605_Ni	995	668	5.474	3.628	S locus F-box protein with the low allelic sequence polymorphism 1-Sf [*Prunus mume*]
Unigene0044727_Nf-vs-Unigene0013907_Ni	741	1197	2.213	5.623	Reticulon-like protein [*Morus notabilis*]
Unigene0045414_Nf-vs-Unigene0076168_Ni	328	465	4.066	5.597	--
Unigene0045862_Nf-vs-Unigene0041640_Ni	1140	1161	37.196	37.298	BnaA03g55340D [*Brassica napus*]
Unigene0045880_Nf-vs-Unigene0003794_Ni	930	975	53.155	85.191	PREDICTED: outer envelope pore protein 24A, chloroplastic-like [*Nelumbo nucifera*]
Unigene0045993_Nf-vs-Unigene0007948_Ni	342	498	1.950	4.776	PREDICTED: uncharacterized protein LOC103955239 [*Pyrus* x *bretschneideri*]
Unigene0046118_Nf-vs-Unigene0000410_Ni	616	705	5.639	7.765	PREDICTED: dirigent protein 19-like [*Populus euphratica*]
Unigene0047070_Nf-vs-Unigene0018079_Ni	524	555	2.386	4.528	--
Unigene0047760_Nf-vs-Unigene0010491_Ni	918	1653	2.664	8.443	PREDICTED: F-box/FBD/LRR-repeat protein At1g13570-like isoform X3 [*Nicotiana tomentosiformis*]
Unigene0048162_Nf-vs-Unigene0066817_Ni	616	1022	2.571	3.381	OSJNBa0009P12.18 [*Oryza sativa* Japonica Group]
Unigene0048211_Nf-vs-Unigene0024703_Ni	1207	676	3.822	4.116	PREDICTED: uncharacterized protein LOC104880697 isoform X2 [*Vitis vinifera*]
Unigene0048353_Nf-vs-Unigene0043035_Ni	1150	1251	108.153	643.581	PREDICTED: plastid-lipid-associated protein, chloroplastic-like [*Citrus sinensis*]
Unigene0048501_Nf-vs-Unigene0017233_Ni	663	879	3.185	14.294	PREDICTED: pentatricopeptide repeat-containing protein At5g27460 [*Vitis vinifera*]
Unigene0048885_Nf-vs-Unigene0030015_Ni	1382	1459	4.062	8.643	PREDICTED: transcription initiation factor TFIID subunit 8 [*Vitis vinifera*]
Unigene0049171_Nf-vs-Unigene0002475_Ni	680	582	2.656	5.706	PREDICTED: uncharacterized aarF domain-containing protein kinase At1g79600, chloroplastic [*Fragaria vesca* subsp. *vesca*]
Unigene0049328_Nf-vs-Unigene0004197_Ni	970	926	4.354	5.088	E3 ubiquitin-protein ligase protein [*Gossypium arboreum*]
Unigene0049362_Nf-vs-Unigene0029495_Ni	370	419	4.131	14.779	fiber protein Fb7 [*Gossypium barbadense*]
Unigene0049880_Nf-vs-Unigene0032785_Ni	1081	1446	3.856	10.644	Caffeoyl-CoA O-methyltransferase 2 [*Triticum urartu*]
Unigene0050106_Nf-vs-Unigene0034130_Ni	735	839	51.719	333.636	hypothetical protein [*Panax quinquefolius*]
Unigene0050208_Nf-vs-Unigene0021045_Ni	1040	941	5.210	5.293	PREDICTED: protein CHROMATIN REMODELING 4 [*Vitis vinifera*]
Unigene0051197_Nf-vs-Unigene0028403_Ni	379	833	1.980	8.027	PREDICTED: sericin 1 [*Cucumis melo*]
Unigene0051829_Nf-vs-Unigene0020009_Ni	776	479	3.330	2.810	PREDICTED: probable tetraacyldisaccharide 4'-kinase, mitochondrial isoform X3 [*Vitis vinifera*]
Unigene0051964_Nf-vs-Unigene0023275_Ni	1024	991	26.051	26.399	PREDICTED: 50S ribosomal protein L12, chloroplastic-like [*Malus domestica*]
Unigene0051967_Nf-vs-Unigene0064529_Ni	321	446	3.809	4.729	PREDICTED: nucleolar protein 56-like [*Nicotiana tomentosiformis*]
Unigene0051976_Nf-vs-Unigene0028115_Ni	307	1596	2.806	5.792	PREDICTED: reticulon-like protein B21-like [*Solanum tuberosum*]
Unigene0052141_Nf-vs-Unigene0030406_Ni	948	902	4.719	1.642	BnaC04g04860D [*Brassica napus*]
Unigene0052203_Nf-vs-Unigene0040101_Ni	1096	1016	7.555	7.641	PREDICTED: E3 ubiquitin-protein ligase RMA1H1-like [*Sesamum indicum*]

Unigene0052466_Nf-vs-Unigene0075926_Ni	732	362	3.151	3.099	PREDICTED: NAC domain-containing protein 21/22-like [*Sesamum indicum*]
Unigene0052552_Nf-vs-Unigene0011381_Ni	1412	1517	8.443	10.679	PREDICTED: 2-aminoethanethiol dioxygenase-like [*Prunus mume*]
Unigene0052792_Nf-vs-Unigene0024949_Ni	820	795	24.500	599.498	PREDICTED: light-regulated protein-like [*Gossypium raimondii*]
Unigene0053088_Nf-vs-Unigene0035625_Ni	963	954	21.641	13.453	PREDICTED: CASP-like protein 5A1 [*Nicotiana sylvestris*]
Unigene0053783_Nf-vs-Unigene0023836_Ni	1558	701	3.264	6.081	PREDICTED: pentatricopeptide repeat-containing protein At1g74900, mitochondrial [*Prunus mume*]
Unigene0053785_Nf-vs-Unigene0022111_Ni	454	419	5.692	6.105	PREDICTED: serine/threonine-protein phosphatase 7 long form homolog [*Glycine max*]
Unigene0053911_Nf-vs-Unigene0031232_Ni	1250	1159	5.291	1.355	PREDICTED: 60 kDa jasmonate-induced protein-like [*Phoenix dactylifera*]
Unigene0054200_Nf-vs-Unigene0079252_Ni	528	358	3.473	3.635	PREDICTED: histone-lysine N-methyltransferase CLF-like isoform X1 *[Malus domestica*]
Unigene0054229_Nf-vs-Unigene0026410_Ni	744	523	3.399	5.062	PREDICTED: F-box protein SKIP23-like [*Eucalyptus grandis*]
Unigene0054277_Nf-vs-Unigene0077511_Ni	1211	1181	38.916	41.682	--
Unigene0054428_Nf-vs-Unigene0001709_Ni	1058	1557	5.936	23.460	embryonic element binding Factor 6 [*Daucus carota*]
Unigene0054549_Nf-vs-Unigene0020232_Ni	705	581	9.144	5.715	Lectin-domain containing receptor kinase A4.2 [*Medicago truncatula*]
Unigene0054558_Nf-vs-Unigene0045470_Ni	794	786	19.283	29.288	PREDICTED: methyl-CpG-binding domain-containing protein 4-like [*Nicotiana tomentosiformis*]
Unigene0055081_Nf-vs-Unigene0005971_Ni	855	635	3.673	3.887	PREDICTED: probable protein phosphatase 2C 23 isoform X3 [*Gossypium raimondii*]
Unigene0055164_Nf-vs-Unigene0037479_Ni	1219	1530	3.146	17.070	PREDICTED: vitellogenin-2 [*Cicer arietinum*]
Unigene0055953_Nf-vs-Unigene0031633_Ni	331	943	2.267	7.661	PREDICTED: U4/U6 small nuclear ribonucleoprotein Prp31-like [*Glycine max*]
Unigene0056041_Nf-vs-Unigene0006860_Ni	2113	1056	2.196	4.844	PREDICTED: F-box protein At2g16365 isoform X1 [*Nicotiana tomentosiformis*]
Unigene0056860_Nf-vs-Unigene0029098_Ni	543	638	12.793	248.563	--
Unigene0057016_Nf-vs-Unigene0006137_Ni	399	367	2.855	3.301	PREDICTED: protein FAR1-RELATED SEQUENCE 5-like [*Beta vulgaris* subsp. *vulgaris*]
Unigene0057125_Nf-vs-Unigene0034542_Ni	1016	1083	86.671	197.435	transcription factor DcERF1 [*Daucus carota*]
Unigene0057493_Nf-vs-Unigene0022161_Ni	1103	1038	8.843	28.230	BnaA07g11550D [*Brassica napus*]
Unigene0057889_Nf-vs-Unigene0066999_Ni	537	626	2.691	5.520	glycerol-3-phosphate acyltransferase [*Camellia sinensis*]
Unigene0057921_Nf-vs-Unigene0002581_Ni	971	932	6.382	18.296	late embryogenesis abundant hydroxyproline-rich glycoprotein [*Arabidopsis thaliana*]
Unigene0058157_Nf-vs-Unigene0076020_Ni	1134	318	2.377	2.681	--
Unigene0058281_Nf-vs-Unigene0063215_Ni	889	823	2.751	2.072	PREDICTED: probable glutamate--tRNA ligase, cytoplasmic [*Nicotiana tomentosiformis*]
Unigene0058296_Nf-vs-Unigene0074380_Ni	309	343	1.889	3.794	Ribosomal RNA large subunit methyltransferase G [*Gossypium arboreum*]
Unigene0059118_Nf-vs-Unigene0025935_Ni	546	997	2.545	259.699	calcium-binding EF hand family protein [*Populus trichocarpa*]
Unigene0059216_Nf-vs-Unigene0018789_Ni	696	314	3.434	3.430	TIP41-like protein [*Gossypium arboreum*]
Unigene0059333_Nf-vs-Unigene0075499_Ni	509	464	2.784	3.288	--
Unigene0060182_Nf-vs-Unigene0024304_Ni	444	371	7.510	3.266	--
Unigene0060543_Nf-vs-Unigene0005833_Ni	499	926	2.395	13.859	TY4B-J: Transposon Ty4-J Gag-Pol polyprotein [*Gossypium arboreum*]
Unigene0061145_Nf-vs-Unigene0032508_Ni	1007	911	6.567	5.221	PREDICTED: vacuolar protein sorting-associated protein 32 homolog 2 [*Cucumis melo*]
Unigene0061447_Nf-vs-Unigene0033257_Ni	1215	1074	7.501	6.810	trypsin-like peptidase domain protein [*Medicago truncatula*]
Unigene0061510_Nf-vs-Unigene0029769_Ni	918	840	25.426	24.574	NEDD8-specific protease 1 [*Morus notabilis*]
Unigene0061542_Nf-vs-Unigene0004490_Ni	491	779	2.717	170.450	--

Unigene0061546_Nf-vs-Unigene0057205_Ni	657	851	3.172	0.106	PREDICTED: pentatricopeptide repeat-containing protein At5g02830, chloroplastic isoform X2 [*Vitis vinifera*]
Unigene0061598_Nf-vs-Unigene0028969_Ni	766	804	0.472	3.181	Mitochondrial transcription termination factor family protein isoform 1 [*Theobroma cacao*]
Unigene0061666_Nf-vs-Unigene0023406_Ni	359	421	2.554	4.690	PREDICTED: probable histone-lysine N-methyltransferase ATXR3 [*Nelumbo nucifera*]
Unigene0061707_Nf-vs-Unigene0002777_Ni	420	543	4.499	8.429	--
Unigene0061711_Nf-vs-Unigene0043731_Ni	915	1015	34.651	38.154	Na(+)-translocating NADH-quinone reductase subunit A [*Gossypium arboreum*]
Unigene0061944_Nf-vs-Unigene0003703_Ni	1385	1418	3.250	42.405	hypothetical protein BVRB_2g041740 [*Beta vulgaris* subsp. *vulgaris*]
Unigene0062190_Nf-vs-Unigene0003959_Ni	922	755	4.551	8.321	PREDICTED: wall-associated receptor kinase 2-like [*Sesamum indicum*]
Unigene0062351_Nf-vs-Unigene0029469_Ni	627	646	17.018	2.709	PREDICTED: uncharacterized protein LOC105640304 [*Jatropha curcas*]
Unigene0062390_Nf-vs-Unigene0024341_Ni	480	575	1.910	5.307	PREDICTED: QWRF motif-containing protein 2 [*Vitis vinifera*]
Unigene0062644_Nf-vs-Unigene0003225_Ni	1020	1025	118.015	209.395	PREDICTED: cucumber peeling cupredoxin-like [*Solanum lycopersicum*]
Unigene0062899_Nf-vs-Unigene0032819_Ni	843	728	5.834	12.574	PREDICTED: RING-H2 finger protein ATL80 [*Gossypium raimondii*]
Unigene0062944_Nf-vs-Unigene0029557_Ni	327	639	1.785	4.354	--
Unigene0062950_Nf-vs-Unigene0042083_Ni	953	881	90.739	35.858	--
Unigene0062957_Nf-vs-Unigene0011558_Ni	349	787	5.175	0.684	PREDICTED: golgin subfamily B member 1-like [*Beta vulgaris* subsp. vulgaris]
Unigene0063121_Nf-vs-Unigene0072707_Ni	602	519	4.939	3.718	PREDICTED: protein trichome birefringence-like 8 [*Nicotiana sylvestris*]

Table S3. The expression (RPKM) of unigenes identified by RNA-sequencing under significant enrichment analysis included GO and KEGG enrichmentsin *Notopterygium incisum* and *Notopterygium franchetii.*

Orthologous	Category	Gene ID	Length		RPKM		Terms
			N. incisum	***N. franchetii***	***N. incisum***	***N. franchetii***	
ORTHOMCL14798		Unigene0064657_Ni,Unigene0014404_Nf	1076	1080	0.083	29.949	GO0008380

ORTHOMCL15032		Unigene0015294_Ni,Unigene0016873_Nf	1418	1480	2.152	1.183	GO0008380
ORTHOMCL12869		Unigene0007638_Ni,Unigene0016874_Nf	1090	1152	0.247	2.460	GO0008380
ORTHOMCL16124		Unigene0025945_Ni,Unigene0020025_Nf	1080	1051	38.766	33.472	GO0031072
ORTHOMCL16135	GO	Unigene0060713_Ni,Unigene0020092_Nf	649	701	1.798	4.519	GO0006083
ORTHOMCL17505		Unigene0033588_Ni,Unigene0027376_Nf	1021	762	16.481	18.343	GO0008380
ORTHOMCL18047		Unigene0035015_Ni,Unigene0030415_Nf	1249	1226	68.047	10.585	GO0008380
ORTHOMCL16216		Unigene0020511_Ni,Unigene0043758_Nf	440	693	5.405	2.727	GO0032300
ORTHOMCL17922		Unigene0029769_Ni,Unigene0061510_Nf	840	918	24.574	25.426	GO0019783
ORTHOMCL17558	Glutathione metabolism	Unigene0050567_Ni,Unigene0027705_Nf	1141	1298	8.68	1.61	KO04624
ORTHOMCL17864		Unigene0053413_Ni,Unigene0029448_Nf	1039	1030	17.62	13.79	KO04624
ORTHOMCL11569	Plant-pathogen interaction	Unigene0020561_Ni,Unigene0003935_Nf	678	1191	6.16	5.48	KO00480
ORTHOMCL16124		Unigene0025945_Ni,Unigene0020025_Nf	1080	1051	38.77	33.47	KO00480
ORTHOMCL13297		Unigene0035012_Ni,Unigene0009122_Nf	993	910	191.88	8.98	KO00480
ORTHOMCL17232		Unigene0036558_Ni,Unigene0025679_Nf	833	744	84.63	65.66	KO00480
ORTHOMCL18122		Unigene0069980_Ni,Unigene0030886_Nf	688	613	6.65	18.9	KO00480
ORTHOMCL15235	Ribosome biogenesis in eukaryotes	Unigene0016160_Ni,Unigene0029802_Nf	701	993	16.964	1.091	KO03008
ORTHOMCL11943		Unigene0040055_Ni,Unigene0004831_Nf	1125	1267	6.661	16.273	KO03008
ORTHOMCL20729		Unigene0064529_Ni,Unigene0051967_Nf	446	321	4.729	3.809	KO03008
ORTHOMCL11146	Transcription factors	Unigene0019446_Ni,Unigene0002620_Nf	1005	1005	7.8665	4.7556	--
ORTHOMCL13242		Unigene0029362_Ni,Unigene0008932_Nf	1218	1218	7.197	1.0038	--
ORTHOMCL17007		Unigene0029761_Ni,Unigene0024420_Nf	1050	1050	65.863	17.122	--
ORTHOMCL20774		Unigene0075926_Ni,Unigene0052466_Nf	732	732	3.099	3.1508	--
ORTHOMCL16173		Unigene0035192_Ni,Unigene0020265_Nf	1279	1279	0	0.0435	--
ORTHOMCL17851		Unigene0064689_Ni,Unigene0029347_Nf	1262	1262	0.1619	12.727	--
ORTHOMCL11581		Unigene0003955_Ni,Unigene0027094_Nf	775	775	22.315	4.7687	--
ORTHOMCL14612		Unigene0042684_Ni,Unigene0013627_Nf	1097	1097	8.2792	15.477	--
ORTHOMCL12079		Unigene0047012_Ni,Unigene0005121_Nf	803	803	0	8.7895	--
ORTHOMCL11075		Unigene0056293_Ni,Unigene0002378_Nf	1815	1815	25.932	20.806	--
ORTHOMCL18712		Unigene0034542_Ni,Unigene0057125_Nf	1016	1016	197.44	86.671	--
ORTHOMCL11417		Unigene0003531_Ni,Unigene0003489_Nf	866	866	15.349	6.5137	--

Table S4 Annotated unigenes in the glutathione metabolism pathway.

	N. incisum		*N. franchetii*	
glutathione reductase (NADPH) [EC:1.8.1.7]	Unigene0068037	Unigene0053616	Unigene0046618	Unigene0041777
	Unigene0053619	Unigene0009368	Unigene0040084	Unigene0018885
	Unigene0053618	Unigene0000439	Unigene0008289	Unigene0008288
	Unigene0053617		Unigene0003813	Unigene0003162

			Unigene0003385	Unigene0003319
isocitrate dehydrogenase	Unigene0064991	Unigene0056538	Unigene0058905	Unigene0045516
[EC:1.1.1.42]	Unigene0064990	Unigene0037330	Unigene0041475	Unigene0035304
	Unigene0064989	Unigene0037329	Unigene0028840	Unigene0028839
	Unigene0056541	Unigene0003312	Unigene0028838	Unigene0028837
	Unigene0056540	Unigene0002622	Unigene0028836	Unigene0028835
	Unigene0056539	Unigene0002026	Unigene0028823	Unigene0028822
			Unigene0028821	Unigene0028820
			Unigene0015850	Unigene0010518
			Unigene0007647	Unigene0001490
			Unigene0001489	Unigene000028
6-phosphogluconate dehydrogenase	Unigene0081144	Unigene0040440	Unigene0055656	Unigene0043359
[EC:1.1.1.44 1.1.1.343]	Unigene0071097	Unigene0040439	Unigene0043315	Unigene0028272
	Unigene0071096	Unigene0007605	Unigene0028271	Unigene0028270
	Unigene0071095	Unigene0005473	Unigene0028269	Unigene0028268
	Unigene0071094	Unigene0005472	Unigene0018223	Unigene0016563
	Unigene0071093	Unigene0004993	Unigene0009263	Unigene0008925
			Unigene0008691	Unigene0007407
			Unigene0003153	
glucose-6-phosphate 1-dehydrogenase	Unigene0067368	Unigene0062452	Unigene0055270	Unigene0053504
[EC:1.1.1.49 1.1.1.363]	Unigene0067367	Unigene0062451	Unigene0051567	Unigene0040073
	Unigene0067366	Unigene0014238	Unigene0039348	Unigene0027832
	Unigene0067365	Unigene0010002	Unigene0027831	Unigene0027830
	Unigene0062456	Unigene0010001	Unigene0027829	Unigene0027827
	Unigene0062455	Unigene0010000	Unigene0027826	Unigene0009284
	Unigene0062454	Unigene0009999	Unigene0009283	
	Unigene0062453	Unigene0009998		
glutathione S-transferase	Unigene0080588	Unigene0048762	Unigene0061549	Unigene0061014
[EC:2.5.1.18]	Unigene0080566	Unigene0048761	Unigene0060989	Unigene0059816
	Unigene0078978	Unigene0043283	Unigene0059769	Unigene0059008
	Unigene0078028	Unigene0043026	Unigene0057837	Unigene0057553
	Unigene0077820	Unigene0043025	Unigene0056773	Unigene0056533
	Unigene0077140	Unigene0043024	Unigene0055527	Unigene0053760
	Unigene0075655	Unigene0043023	Unigene0053602	Unigene0051188
	Unigene0066993	Unigene0040668	Unigene0049847	Unigene0049813
	Unigene0053414	Unigene0040667	Unigene0049026	Unigene0048910
	Unigene0053413	Unigene0040666	Unigene0048556	Unigene0046658
	Unigene0053412	Unigene0037957	Unigene0045285	Unigene0045017
	Unigene0053411	Unigene0037956	Unigene0044170	Unigene0043973
	Unigene0048764	Unigene0034219	Unigene0042316	Unigene0042240
gamma-glutamyltranspeptidase / glutathione				
hydrolase	Unigene0069282	Unigene0055220	Unigene0060571	Unigene0058238
[EC:2.3.2.2 3.4.19.13]	Unigene0069281	Unigene0054170	Unigene0048612	Unigene0039698
	Unigene0069280	Unigene0035636	Unigene0021387	Unigene0013321

	Unigene0069279	Unigene0027758		
	Unigene0069278	Unigene0018157		
	Unigene0069277	Unigene0018156		
	Unigene0069276	Unigene0008183		
	Unigene0069275	Unigene0008182		
	Unigene0069274	Unigene0008181		
	Unigene0069273	Unigene0008180		
	Unigene0055224	Unigene0008179		
	Unigene0055223	Unigene0008178		
	Unigene0055222	Unigene0008177		
	Unigene0055221			
glutathione synthase	Unigene0062364	Unigene0062362	Unigene0023738	
[EC:6.3.2.3]	Unigene0062363	Unigene0062361		
glutamate--cysteine ligase catalytic subunit	Unigene0051296	Unigene0001767	Unigene0048692	Unigene0041902
[EC:6.3.2.2]	Unigene0051295	Unigene0039501	Unigene0041672	Unigene0011934
glutathione peroxidase	Unigene0081311	Unigene0039500	Unigene0049858	Unigene0049857
[EC:1.11.1.9]	Unigene0050568	Unigene0039499	Unigene0048668	Unigene0046812
	Unigene0050567	Unigene0037119	Unigene0027706	Unigene0027705
	Unigene0050566	Unigene0037118	Unigene0027704	Unigene0019204
	Unigene0050565	Unigene0037117	Unigene0018038	Unigene0017822
	Unigene0050564	Unigene0037116	Unigene0017748	Unigene0017744
	Unigene0050563	Unigene0036702	Unigene0013923	Unigene0013922
	Unigene0047559	Unigene0036701	Unigene0012478	Unigene0010840
	Unigene0047558	Unigene0031589	Unigene0010839	Unigene0010687
	Unigene0047557	Unigene0031588	Unigene0010686	Unigene0008495
	Unigene0047556	Unigene0031587	Unigene0001858	
	Unigene0047555	Unigene0028922		
	Unigene0047554	Unigene0028480		
	Unigene0047553	Unigene0047552		

Table S5 Annotated unigenes in the Plant-pathogen interaction pathway.

	N. incisum			*N. franchetii*		
CDPK	Unigene0078771	Unigene0076596	Unigene0074962	Unigene0061479	Unigene0060452	Unigene0017816
	Unigene0074507	Unigene0072892	Unigene0072894	Unigene0058419	Unigene0058379	Unigene0057004
	Unigene0072893	Unigene0069326	Unigene0069325	Unigene0051350	Unigene0048510	Unigene0043619
	Unigene0069324	Unigene0069323	Unigene0069322	Unigene0040405	Unigene0038220	Unigene0036076
	Unigene0069321	Unigene0069320	Unigene0065960	Unigene0034978	Unigene0032242	Unigene0032233
	Unigene0065959	Unigene0065958	Unigene0065733	Unigene0031998	Unigene0031997	Unigene0031994
	Unigene0065119	Unigene0065118	Unigene0065117	Unigene0030434	Unigene0030433	Unigene0030432
	Unigene0065116	Unigene0061954	Unigene0061953	Unigene0028294	Unigene0028293	Unigene0028292
	Unigene0061952	Unigene0061961	Unigene0061102	Unigene0028291	Unigene0024826	Unigene0022006
	Unigene0061101	Unigene0061100		Unigene0020017	Unigene0019241	Unigene0018354
Rboh	Unigene0007628	Unigene0001257	Unigene0000488	Unigene0056126	Unigene0046157	Unigene0002989
	Unigene0012525	Unigene0009970	Unigene0009501	Unigene0045455	Unigene0040249	Unigene0040010
	Unigene0018711	Unigene0018710	Unigene0015222	Unigene0039809	Unigene0039046	Unigene0038639
	Unigene0027567	Unigene0027566	Unigene0023284	Unigene0033948	Unigene0032618	Unigene0032617
	Unigene0041741	Unigene0041746	Unigene0040146	Unigene0016600	Unigene0016599	Unigene0010382
	Unigene0057347	Unigene0057346		Unigene0004890		
NOS	Unigene0049505	Unigene0049504	Unigene0049503	Unigene0011105		
	Unigene0049502					
FLS2	Unigene0077965	Unigene0076049	Unigene0021380	Unigene0061564	Unigene0048877	Unigene0013732
MBAK1HKK1	Unigene0072132	Unigene0072131	Unigene0072130	Unigene0020093		
	Unigene0072129	Unigene0072128	Unigene0018475			
	Unigene0010193					
MEKK1	Unigene0080237	Unigene0079599	Unigene0079517	Unigene0046092	Unigene0043605	Unigene0024004
	Unigene0048961	Unigene0048960	Unigene0048959	Unigene0023334	Unigene0002986	
	Unigene0048958	Unigene0048957	Unigene0038650			
	Unigene0038649	Unigene0038648	Unigene0023385			
	Unigene0002200					
MKK1/2	Unigene0046028	Unigene0046027		Unigene0053280	Unigene0045018	
MKK4/5	Unigene0040097	Unigene0040096	Unigene0024914	Unigene0008487	Unigene0007664	
	Unigene0020960	Unigene0006965				
WRKY25	Unigene0068488	Unigene0068487	Unigene0068486	Unigene0019038	Unigene0019037	Unigene0019036
	Unigene0068484	Unigene0068483	Unigene0068482	Unigene0019035	Unigene0011125	Unigene0010879
	Unigene0068480	Unigene0068479	Unigene0068478	Unigene0010878		
	Unigene0068477	Unigene0068476	Unigene0068475			
	Unigene0068474	Unigene0068473	Unigene0068471			
	Unigene0068470	Unigene0068469	Unigene0068468			
	Unigene0054416	Unigene0054414	Unigene0054411			
	Unigene0054410	Unigene0054409	Unigene0041442			
	Unigene0041441	Unigene0025394	Unigene0024563			
	Unigene0078502	Unigene0068489				
WRKY29	Unigene0034809	Unigene0034808	Unigene001242	Unigene0058174	Unigene0037917	Unigene0008932

	Unigene0034807	Unigene0029365	Unigene0029364	Unigene0008931		
	Unigene0029363	Unigene0029361	Unigene0028980			
FRK1	Unigene0025563					
NHO1	Unigene0057867	Unigene0016441	Unigene0001619	Unigene0050314	Unigene0048951	Unigene0045994
				Unigene0044676	Unigene0044177	Unigene0017742
				Unigene0007514		
PR1	Unigene0036559	Unigene0036558	Unigene0024645	Unigene0052102	Unigene0049037	Unigene0042951
	Unigene0018981	Unigene0011824	Unigene0000951	Unigene0042473	Unigene0037667	Unigene0036386
				Unigene0025679	Unigene0014396	
Pti4	Unigene0040283	Unigene0040282	Unigene0040281	Unigene0017929	Unigene0017928	
	Unigene0040280	Unigene0040279	Unigene0040278			
	Unigene0040277	Unigene0036658	Unigene0036657			
Pti6	Unigene0079066	Unigene0032740	Unigene0006101	Unigene0033998	Unigene0033997	
Pti1	Unigene0062242	Unigene0062241	Unigene0062240	Unigene0030561	Unigene0030560	Unigene0030274
	Unigene0062239	Unigene0062238	Unigene0062237	Unigene0030273	Unigene0030271	Unigene0016090
	Unigene0059764	Unigene0059763	Unigene0058929	Unigene0000581		
	Unigene0058928	Unigene0058927	Unigene0058926			
	Unigene0058925	Unigene0058924	Unigene0058923			
	Unigene0058922	Unigene0058921	Unigene0058919			
	Unigene0058918	Unigene0058917	Unigene0058916			
	Unigene0026555	Unigene0020744	Unigene0015807			
	Unigene0012239	Unigene0012238	Unigene0006845			
	Unigene0005077	Unigene0076931	Unigene0062243			
RIN4	Unigene0028754	Unigene0028753	Unigene0024122	Unigene051267	Unigene0017173	Unigene0017172
	Unigene0024121	Unigene0020561	Unigene0019246	Unigene0017171	Unigene0017170	Unigene0009591
	Unigene0032379	Unigene0032378		Unigene0003935		
RPM1	Unigene0076096	Unigene0075601	Unigene0072070	Unigene0050597	Unigene0049275	Unigene0005036
	Unigene0072068	Unigene0052975	Unigene0045352	Unigene0043774	Unigene0041234	Unigene0039040
	Unigene0029661	Unigene0029660	Unigene0019198	Unigene0020711	Unigene0020018	Unigene0017998
	Unigene0018845	Unigene0014378	Unigene0013599	Unigene0014564	Unigene0002747	
	Unigene0013058	Unigene0012608	Unigene0007725			
	Unigene0079578	Unigene0077211				
RPS2	Unigene0073803	Unigene0072314	Unigene0072313	Unigene0033636	Unigene0025450	Unigene0002474
	Unigene0072311	Unigene001738	Unigene0071736	Unigene0001676		
	Unigene0019924	Unigene0017693	Unigene0008039			
	Unigene0004237	Unigene0000535	Unigene0077763			
	Unigene0074526					
RPS1	Unigene0073912	Unigene0073911	Unigene0073910	Unigene0032259	Unigene0032258	Unigene0032257
	Unigene0073909	Unigene0073908	Unigene0073907	Unigene0001566		
	Unigene0073906	Unigene0041340	Unigene0016699			
	Unigene0016698	Unigene0016697	Unigene0016696			
	Unigene0073914	Unigene0073913				
SGT1	Unigene061825	Unigene0061824	Unigene0017497	Unigene0051512	Unigene0023256	Unigene0023255
	Unigene0015910	Unigene0006519	Unigene0006223			

	Unigene0061827	Unigene0061826				
EDS1	Unigene0059559	Unigene0059558	Unigene0059560	Unigene0042830	Unigene0038958	Unigene0017264
	Unigene0059561	Unigene0059557	Unigene0015503	Unigene0014458	Unigene0014457	Unigene0002419
	Unigene0034297	Unigene0034296	Unigene0034295	Unigene		
	Unigene0021766	Unigene0021675	Unigene0019610			
	Unigene0018414	Unigene0018397	Unigene0015505			
	Unigene0015504					
RAR1	Unigene0025946	Unigene0025945		Unigene0020025		
SGT1	Unigene0061825	Unigene0061824	Unigene0017497	Unigene0051912	Unigene0023256	Unigene0023255
	Unigene0015910	Unigene0006519	Unigene0006223			
	Unigene0061827	Unigene0061826				
SHSP90	Unigene0072364	Unigene0062067	Unigene0000270	Unigene0060062	Unigene0062414	Unigene0048977
	Unigene0062066	Unigene0062065	Unigene0060768	Unigene0046575	Unigene0020586	Unigene0011910
	Unigene0060767	Unigene0060766	Unigene0060765	Unigene0011909	Unigene0011831	Unigene0011830
	Unigene0060764	Unigene0060414	Unigene0060413	Unigene0011829	Unigene0002461	Unigene0059259
	Unigene0060412	Unigene0060411	Unigene0029991	Unigene0054679	Unigene0050826	Unigene0047952
	Unigene0029990	Unigene0019811	Unigene0018170	Unigene0047192	Unigene0046190	Unigene0040183
	Unigene0012162	Unigene0011680	Unigene0011679	Unigene0035680	Unigene0034680	Unigene0033182
	Unigene0006733	Unigene0003069	Unigene0002286	Unigene0033179	Unigene0033178	Unigene0033177
	Unigene0002285	Unigene0001442	Unigene0000965	Unigene0033176	Unigene0033175	Unigene0033174
				Unigene0033171	Unigene0033170	
WRKY1/2	Unigene0052626	Unigene0052625	Unigene005624	Unigene0022912	Unigene0022911	Unigene0007817
	Unigene0052623	Unigene0004337	Unigene0001875	Unigene0007870		
	Unigene0000264	Unigene0075015	Unigene0060387			
	Unigene0060386	Unigene0060385	Unigene0060384			
	Unigene0060383	Unigene0060382	Unigene0050209			
	Unigene0050208	Unigene0050207	Unigene0050206			
	Unigene0002093					
CNGCs	Unigene0080508	Unigene0079323	Unigene0048040	Unigene0048766	Unigene0042160	Unigene0002147
	Unigene0076163	Unigene0075305	Unigene0073848	Unigene0038308	Unigene0038307	Unigene0034359
	Unigene0073847	Unigene0073846	Unigene0071313	Unigene0032169	Unigene0032168	Unigene0030886
	Unigene0071312	Unigene0071311	Unigene0071310	Unigene0030885	Unigene0029625	Unigene0018173
	Unigene0071309	Unigene0071308	Unigene0069983	Unigene0018172	Unigene0018171	Unigene0014736
	Unigene0069980	Unigene0066470	Unigene0062911	Unigene0014735	Unigene0014681	Unigene0013955
	Unigene0062910	Unigene0062164	Unigene0062163	Unigene0013954	Unigene0006942	Unigene0002148
	Unigene0062162	Unigene0062161	Unigene0059889			
	Unigene0059888	Unigene0049658	Unigene0049657			
	Unigene0049656	Unigene0048042	Unigene0048041			
CaM/CML	Unigene0057851	Unigene0048386	Unigene0048385	Unigene0061116	Unigene0059213	Unigene0030402
	Unigene0048384	Unigene0048383	Unigene0048382	Unigene0058775	Unigene0058641	Unigene0056562
	Unigene0048381	Unigene0045063	Unigene0045062	Unigene0053339	Unigene0051163	Unigene0050915
	Unigene0045061	Unigene0045060	Unigene0045059	Unigene0050498	Unigene0050433	Unigene0050203
	Unigene0039872	Unigene0039871	Unigene0039870	Unigene0049130	Unigene0047115	Unigene0046883
	Unigene0035012	Unigene0034125	Unigene0029878	Unigene0043987	Unigene0042566	Unigene0041533

Unigene0022048	Unigene0021342	Unigene0020375	Unigene0041098	Unigene0040940	Unigene0040923
Unigene0019393	Unigene0019392	Unigene0014573	Unigene0038802	Unigene0036447	Unigene0035885
Unigene0010604	Unigene0010603	Unigene0010334	Unigene0035884	Unigene0035627	Unigene0034989
Unigene0007401	Unigene0006875		Unigene0034746	Unigene0034681	

Table S6 Characteristics of 17 SSR markers in this study.

Locus	Primer sequences (5′-3′)	Repeat motif	Allele size rang (bp)	Ta (°C)
**11140**	F: CCTCCCCAGCATTAGTCTGA	(AAG)5	202-238	53
	R: GCTCAAATCTCCTCCATCACA			

**12542**	F: TTGTCTTCTGCGGTTGTACG	(GTG)5	204-210	55
	R: ATTCTCCTTCTCTCCGCCAT			
**15567**	F: GATCTCAGATGAGGAAGCGG	(GAA)6	245-263	55
	R: TCAACGCTCGATTTCATCAG			
**15401**	F: GAGGGTTTTACTGCAGAGACAGA	(AGA)5	177-182	54
	R: TCGCACTCAATCATGCTACC			
**15156**	F: CGCAGCAACAACAACAAACT	(ACA)5	152-176	55
	R: TAAGTAGCCGGAGCCTTGTC			
**14926**	F: TTGCTTCGACGAATCCATAG	(TGC)6	243-261	55
	R: GGCAAAACAATCCTCAGTCAA			
**14424**	F: GTGAATGTTGTGGACCTCCC	(CCT)5	243-249	55
	R: GGCAGGATACGAAGCCTAGA			
**14261**	F: TCCCATCCATCTCTTACAAAA	(TTC)5	177-183	54
	R: ATTTTGAAAAATCCCCGACC			
**14072**	F: GGTCGGGTCTCACACAAAAT	(AGA)5	205-240	55
	R: TGGATACTTTTTCTGTTTGGTCTTT			
**16151**	F: TTTAAGGAATGACCAACGGC	(ATC)8	243-288	57
	R: AGTGGAGGAGGATCATGGTG			
**16678**	F: ACATGTCCTCCATGGACTGG	(TCA)7	231-240	54
	R: GCCTAGTAAGCGTCGTCGTC			
**18018**	F: CCATGGCCTTTGCTCATTAC	(TCT)5	226-238	54
	R: CGATGAAGAAGCTTGGGAAA			
**17078**	F: AGGTGGAAATGTCAAGTGGC	(CTG)5	267-279	56
	R: CACCGTCCTCTGCTTCTTTC			
**18934**	F: GCCATCACTAGCCTTTGGAG	(CAT)5	241-265	54
	R: GTCGCTTTGCCCGTAAATAA			
**19210**	F: GTTGTGGAGGAGAAGGCAAA	(AGA)6	263-281	55
	R: CAACTGTCTCCTCCGCTTTC			
**20218**	F: ATGGGTGTGGAAGATTCAGC	(ATT)5	101-104	55
	R: TATTCGCACTCGAAAATCCC			
**15648**	F: TTGTGAGGAATTAGCTGGGG	(CTT)6	244-262	56
	R: GGGTCCAGTCTCAGCTACCA			

Table S7 Comparison of genetic diversity between *Notopterygium incisum* and *Notopterygium franchetii* based on 17 SSR loci.

	N		*Ho*		H_E		*I*	
Locus	*N. incisum*	*N. franchetii*	*N. incisum*	*N. franchetii*	*N. incisum*	*N. franchetii*	*N. incisum*	*N. franchetii*
11140	1	2	0	0.15	0	0.365	0	0.51

12542	2	1	0.16	0	0.224	0	0.378	0
15567	3	2	0.12	0.25	0.584	0.265	0.968	0.404
15401	2	1	0.12	0	0.18	0	0.318	0
15156	2	2	0.24	0.15	0.296	0.195	0.518	0.326
14926	1	3	0	0.2	0	0.435	0	0.747
14424	2	2	0.04	0.35	0.164	0.325	0.262	0.467
14261	2	1	0.04	0.25	0.036	0.125	0.065	0.173
14072	2	2	0.09	0.05	0.29	0.17	0.432	0.255
16151	3	1	0.41	0	0.458	0	0.819	0
16678	1	2	0	0.75	0	0.375	0	0.52
18018	2	2	0.16	0.05	0.34	0.125	0.578	0.173
17078	2	2	0.42	0.888	0.291	0.487	0.434	0.68
18934	1	2	0.08	0.25	0.128	0.205	0.2	0.328
19210	1	1	0.04	0	0.1	0	0.165	0
20218	1	1	0.04	0	0.036	0	0.065	0
15648	1	2	0.185	0.1	0.296	0.19	0.504	0.317
Mean	1.765	1.529	0.126	0.202	0.201	0.192	0.336	0.288

N, number of different alleles; *Ho*, Observed Heterozygosity; H_E, Expected Heterozygosity; *I*, Shannon's Information Index.

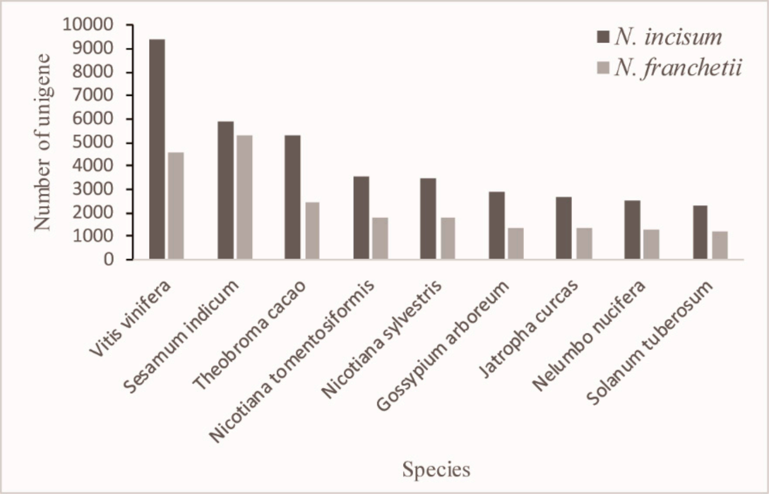

Figure. S1 Top-Hit species distribution of BLASTx matches to unigenes.

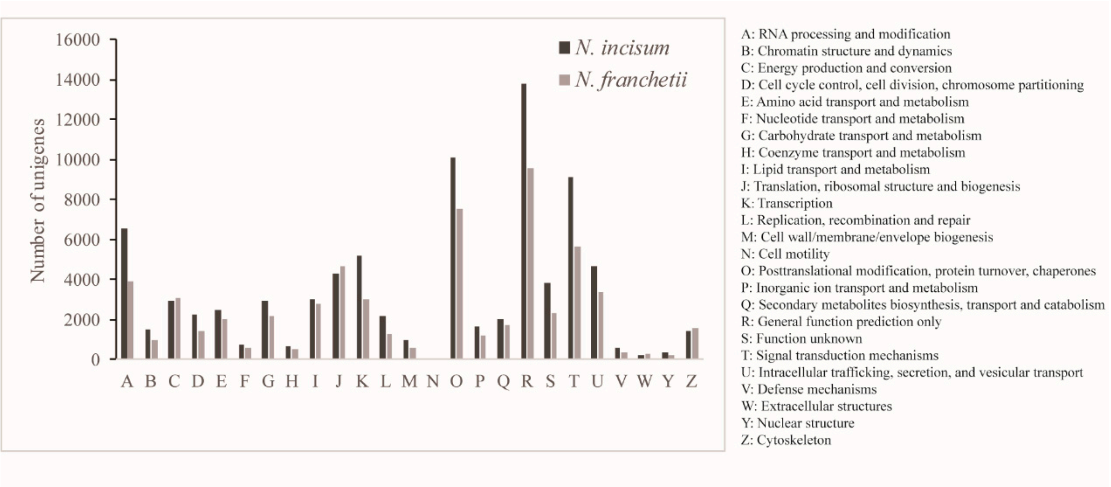

Figure. S2 KOG function classification of unigenes.

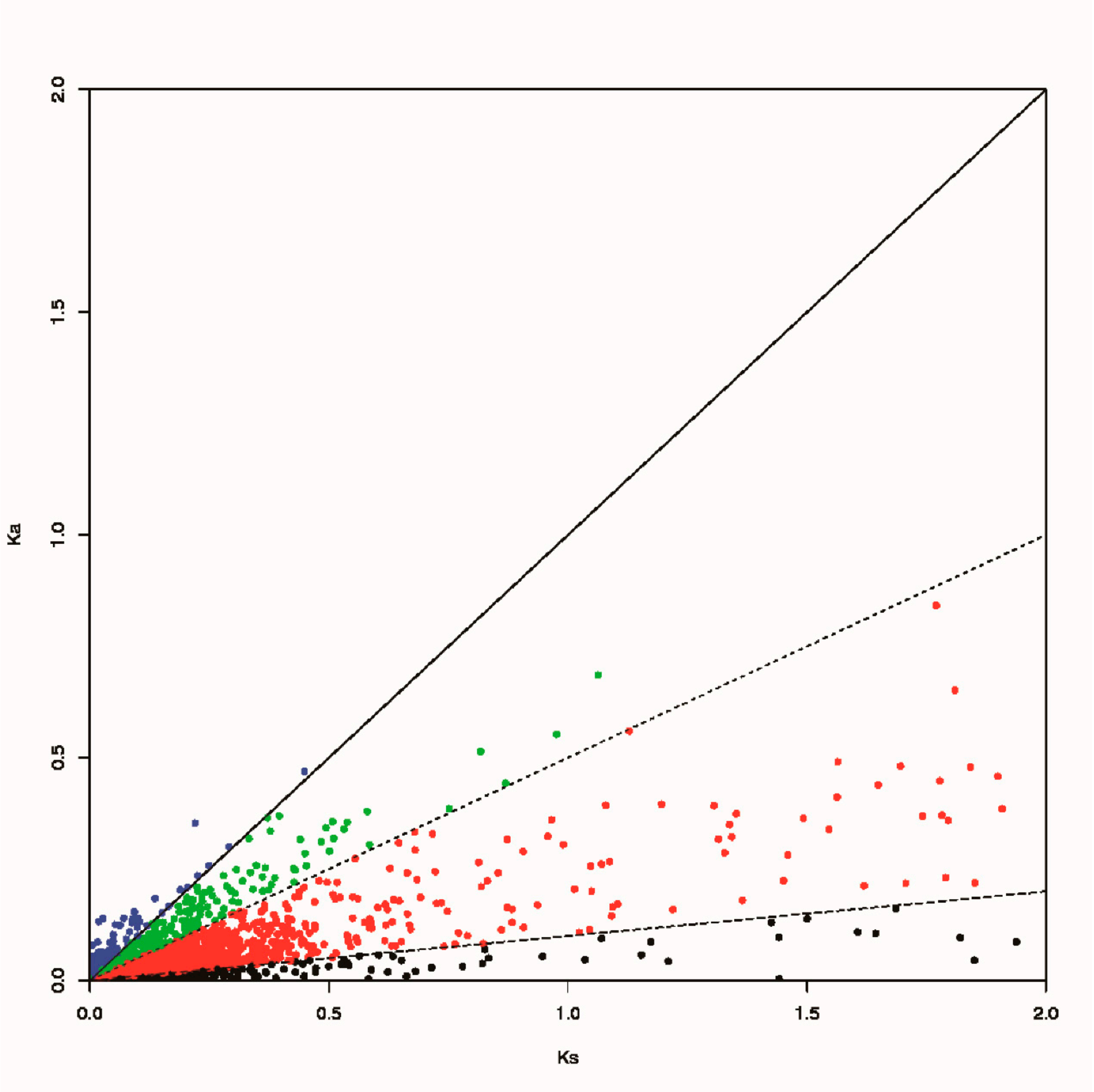

Figure S3. A total of 3823 pairs of single-copy orthologous genes were found and used to calculate Ka/Ks ratios. Of these orthologs, 381 pairs with a Ka/Ks value > 1 were found indicating positive selection (blue dots), and 857 had a Ka/Ks ratio between 0.5 and 1, indicating weak purifying selection (green dots).

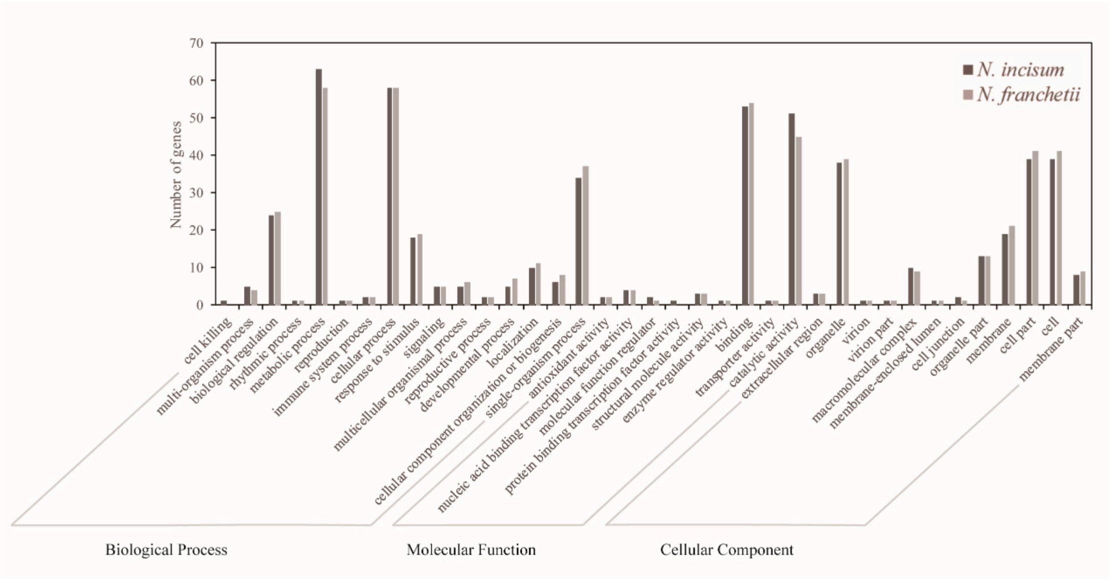

Figure S4. Gene Ontology (GO) distributions of positively selected genes.

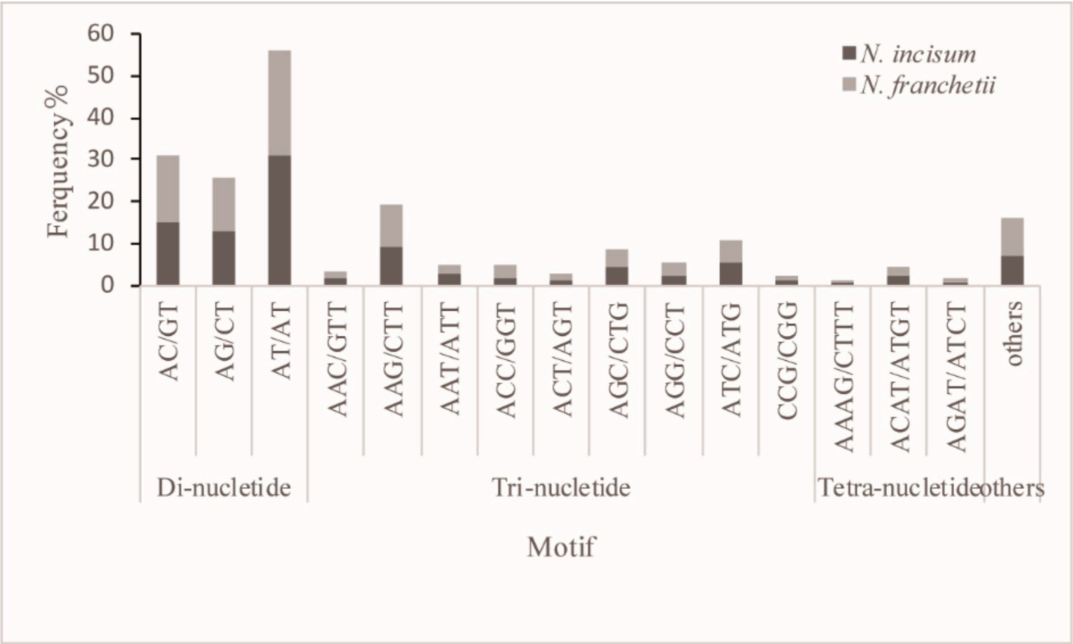

Figure. S5 Distribution of different SSR repeat motif types.

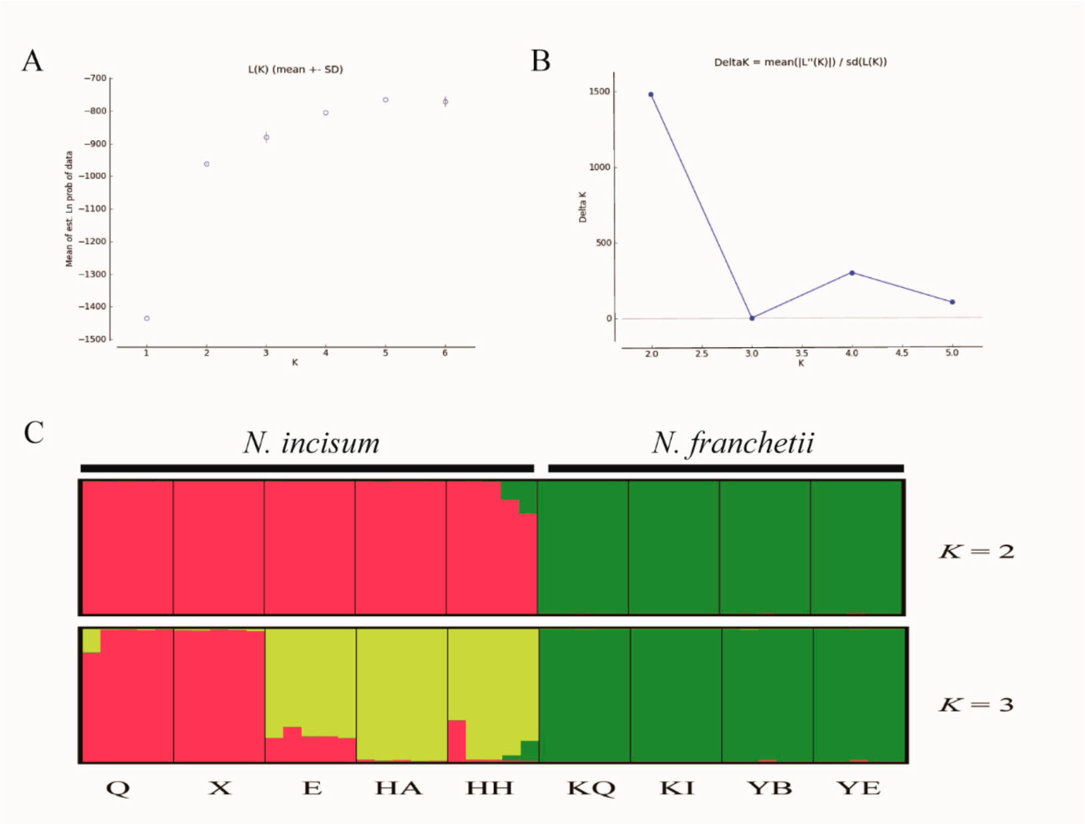

Figure. S6 Bayesian inference analysis of microsatellite data for determining the most likely number of cluster (K) for the two species. The distribution of the likelihood L (K) values (A) and ΔK values (B) are presented for 1-6 (20 replicates). STRUCTURE plots are presented for best K = 2 and K = 3, respectively.
